# Regional Ground-Based IoT Solar Irradiance Monitoring: A Multi-Site Study Across Mountain, Rural, and Urban Environments

**DOI:** 10.3390/s26185781

**Published:** 2026-09-11

**Authors:** Dejan Vujičić, Dušan Marković, Pranay Obla Anandbabu, Shrihari Rajeev Kulkarni, Zoran Stamenković, Siniša Ranđić

**Affiliations:** 1 Faculty of Technical Sciences Čačak, University of Kragujevac, 32102 Čačak, Serbia; dejan.vujicic@ftn.kg.ac.rs (D.V.); sinisa.randjic@ftn.kg.ac.rs (S.R.); 2 Faculty of Agronomy Čačak, University of Kragujevac, 32102 Čačak, Serbia; dusan.markovic@kg.ac.rs; 3 Viterbi School of Engineering, University of Southern California, Los Angeles, CA 90089, USA; oblaanan@usc.edu; 4 School of Computer Science and Engineering, Vellore Institute of Technology, Vellore 632014, India; kshrihari0611@gmail.com; 5 Synopsys Armenia Educational Department, National Polytechnic University of Armenia, Yerevan 0026, Armenia

**Keywords:** solar irradiance, IoT, low-cost sensors, ground-based monitoring, NASA POWER, machine learning, sky view factor, edge computing, ESP32

## Abstract

In this paper, the solar irradiance is investigated in a part of central Serbia, where a low-cost IoT sensor network was deployed at three locations: a mountain slope, an open field near a village, and an obstructed position in the city center. All three locations are in the same NASA POWER grid cell. After multi-stage quality control, 26,145 valid daytime records were compared to the satellite reference. The satellite assigns identical values to all three positions but the measured mean daytime irradiances are 267.7, 351.5 and 19.6 W/m^2^, respectively. The rural station is in the best agreement with the reference (R^2^ = 0.567); the mountain station suffers from a persistent shading bias (MBE = −138.9 W/m^2^); the signal at the urban station is attenuated by surrounding buildings and vegetation by a factor of ~12. Also, 25 regression models (gradient boosting, recurrent, convolutional, fully connected and graph-based) were trained on the 25-year monthly NASA POWER record for the same cell. XGBoost obtained R^2^ = 0.998, the hybrid TCN-GNN R^2^ = 0.968 and a plain ReLU network R^2^ = 0.912, and a seasonal model with calendar features was used to reconstruct the satellite reference for two months of 2026 not yet available in the archive. The deployed hardware, network and energy behavior are documented quantitatively: per-site link delivery ratios of 96.8%, 83.5% and 58.2%, the photovoltaic harvesting record of the nodes, and the absence of any energy-aware transmission scheduling. Two of the trained models were compiled for the ESP32 nodes and measured on the deployment hardware: a depth-limited gradient-boosted corrector runs in 46.3 µs using 11.1 kB of flash, and an INT8 fully connected network in 138.1 µs using 3.0 kB, while the graph hybrid cannot be converted for microcontroller execution at all. This defines an edge–cloud partition in which local inference performs bias correction and fault detection while the cloud tier retains the heavy models and periodic retraining. To the best of the authors’ knowledge, this is the first multi-site ground-based IoT irradiance record for central Serbia with such different terrain types.

## 1. Introduction

Ground measurements of solar irradiance are needed for two related purposes: they form the basis for the validation of satellite-derived datasets and they provide the training data required to train machine learning forecasting models to ensure their output reflects actual local conditions. Satellite products such as NASA POWER [1] provide continuous spatial coverage, but their grid resolution of 0.5° × 0.5° (about 2500 km^2^ per cell) is too coarse to capture the variations introduced by terrain, vegetation or the geometry of a built-up street at a given point. In mountain valleys, the difference can be large, as ridge shading, radiation fog, and locally developed convective cloud all act on scales well below the grid resolution.

Published work on solar irradiance in the Balkan region is mostly based on gridded datasets, mainly NASA POWER and PVGIS [2] or on numerical weather prediction output, again at the resolution of half a degree or coarser. Ground validation studies for the Western Balkans are scarce and almost non-existent for Serbia. The national meteorological service operates professional stations, but they are insufficient for characterizing the micro-scale irradiance environment relevant for distributed photovoltaics and building-integrated systems.

A second gap concerns the instrumentation. Much low-cost irradiance monitoring stops at the acquisition front end: the nodes collect data, ship it to a server, and all analysis is performed offline, so the sensing hardware is interchangeable with any commercial datalogger. Which part of the processing chain can execute on the node, and at what cost in latency and memory, is rarely measured.

In this paper we present solar irradiance data obtained with a low-cost IoT sensor network at three quite different locations around Čačak. The measurements reveal the strong dependence of the local irradiance regime on the site and quantify the difference between ground values and the NASA POWER reference for each terrain type. The dataset presented in this paper is of supporting nature: it characterizes the local solar environment and demonstrates the value of ground-based IoT monitoring for satellite-based irradiance prediction in complex terrain.

The aims of this work are (1) to present and validate an autonomous, low-cost solar irradiance measurement system deployed across mountain, rural, and urban settings; (2) to quantify site-specific irradiance regimes and their departure from the NASA POWER satellite reference as direct evidence of sub-grid variability; (3) to provide an open-source benchmark dataset alongside 25 locally retrained regression models spanning ensemble, recurrent, convolutional, and graph-based architectures; (4) to document the network topology, HTTP ingestion protocol, link reliability, and autonomous photovoltaic energy budget of the deployed nodes; and (5) to establish, via direct benchmarking on the ESP32 microcontroller, which model architectures can execute at the edge versus those relegated to the cloud, formalizing a hierarchical intelligence pipeline where local lightweight inference performs real-time bias correction and anomaly detection while cloud resources handle computationally heavy forecasting and periodic model retraining.

## 2. Related Work

The satellite and reanalysis irradiance products have been extensively evaluated against ground networks. The methodology of the NASA POWER project [1] has been described and typical hourly uncertainties of 15–20% have been reported for mid-latitude land sites, while PVGIS [2] and the National Solar Radiation Data Base [3] employ similar satellite-to-surface modeling chains. Urraca et al. [4] compared global horizontal irradiance from the ERA5 and COSMO-REA6, reanalyzed with ground and satellite data over Europe, and found biases that vary from region to region, making a case for local validation wherever a product is used quantitatively. Gueymard [5] systematized the statistical indicators for such comparisons (RMSE, MBE and correlation-based measures), and his recommendations are followed here in the validation. This whole work is based on reliable ground stations, and they are not evenly distributed—South-East Europe has few of them.

On the modeling side, classical parametric approaches to irradiance forecasting have been largely supplanted by machine learning. Voyant et al. [6] reviewed the evolution from regression baselines to ensemble tree methods and neural networks. Yagli et al. [7] drew similar conclusions from comparing 68 algorithms for hourly forecasting. The same tendency is visible outside the solar domain: Alim et al. [8] compared ARIMA with XGBoost on epidemiological time series in mainland China and found that XGBoost gave lower MAE, RMSE and MAPE on both the training and the test data, mainly because gradient boosting follows irregular and nonlinear patterns more easily than a linear model. Gradient boosting as implemented in XGBoost [9] is a robust model for tabular meteorological data.

The most common recurrent choices for sequence models are long short-term memory networks [10] and the simpler gated recurrent unit [11]. Waqas and Humphries [12] studied the RNN, LSTM and GRU variants used in hydrological time series prediction; those models well describe the nonlinear relations and long-term dependencies of such systems, even when the data are sparse or incomplete, but their black-box character makes the interpretation difficult. Amalou et al. [13] used GRU, LSTM and simple RNN for household energy consumption data from a smart grid and found that the GRU gives a good balance between computational cost and accuracy, as it keeps the key sequence information with a simpler structure than the LSTM. Zhang et al. [14] proposed eGRU, an adaptation of the GRU for extreme events, which uses different hidden states for normal and extreme behavior and outperformed the standard GRU, LSTM and other recurrent models on several multivariate forecasting problems. Balti et al. [15] used a BiGRU encoder–decoder with a temporal attention layer and achieved better accuracy than ARIMA, PROPHET and several deep learning baselines in univariate and multivariate tasks.

Convolutional architectures process the sequence in parallel and not sequentially. Shahid et al. [16] examined 1D and 2D convolutional networks for real-time classification of sensor time series and found that shallow networks of either kind achieve comparable accuracy, but the 1D one requires far less computation. Temporal convolutional networks [17] are based on dilated causal convolutions and have been shown to be useful for energy-related series: TCNs have been found to outperform LSTMs in accuracy and robustness over a range of hyperparameter settings, for national electricity demand and for electric vehicle charging data [18]. In time series anomaly detection, he and Zhao [19] used TCNs and modeled the prediction errors with a multivariate Gaussian. Tang et al. [20] proposed channel attention mechanism to TCN (CA-TCN) and showed it performed better than random forests, CNNs, Bi-LSTMs and transformers for satellite image time series classification.

Hybrid architectures combining convolutional feature extraction and recurrent temporal modeling have demonstrated good performance for financial and commodity forecasting. A CNN-LSTM model was proposed by Livieris et al. [21] for gold prices forecasting. Convolutional layers are used to extract features from the noisy series and LSTM layers are used to model the temporal dependence. The errors were clearly lower than the standalone LSTM, SVR and random forest, especially in multi-step prediction.

Widiputra et al. [22] extended the idea and built a multivariate CNN-LSTM that simultaneously predicts multiple financial series and achieved lower RMSE than individual CNN and LSTM models during the volatile COVID-19 period. Zha et al. [23] proposed to combine CNN-LSTM with recursive prediction strategy for monthly gas production and obtained a MAPE of 7.7%. Chen et al. [24] found that Bayesian-tuned CNN-GRU improves the accuracy and generalization in oil production forecasting, because CNN extracts local and higher-level features while GRU handles long-term dependence with less training effort than LSTM. Graph neural networks, based on the graph convolution operator of Kipf and Welling [25] and on spatio-temporal designs such as Graph WaveNet [26], extend sequence modeling to structured dependencies between time steps or sensor nodes: Tang et al. [27] combined GNNs with GRUs and attention (GRN) for interpretable anomaly detection in industrial control systems, and Li et al. [28] combined adaptive graph convolution with a gate-enhanced neural ODE (RST-LTG) and outperformed 19 other models on four benchmark datasets.

A further set of studies has investigated whether low-cost illuminance sensors can replace pyranometers. Nava-Pintor et al. [29] compared BH1750 and VEML7700 with a reference solar radiation sensor and showed that the sensors can be used for irradiance estimation with a correction based on regression. The lux-to-irradiance conversion framework used in the present study was given by Michael et al. [30] and the field performance of the BH1750FVI was reported by Silveira et al. [31] against a reference meteorological station. In general, it can be concluded that such sensors reach an absolute accuracy of about ±25–30%, which is far from metrological grade but sufficient for relative comparisons, gap detection and network densification at a small fraction of pyranometer cost.

In cities the controlling variable is the geometry of the visible sky. Hofierka and Zlocha [32] developed a 3-D solar radiation model for city conditions, where the sky view factor determines the attenuation of global irradiance in street canyons; reductions of 30–90% are typical when the sky view factor is less than 0.5. The results are used below to interpret urban measurements.

For Serbia in particular, the published assessments mainly concern resource potential at national scale, e.g., the review of concentrating solar power prospects by Pavlović et al. [33], while systematic ground measurement remains limited to the stations of the national hydrometeorological service [34]. To the authors’ knowledge, this type of multi-site campaign with low-cost instrumentation across contrasting terrain has not been published for this region.

## 3. Measurement System

### 3.1. Sensor Hardware

**Figure 1:**
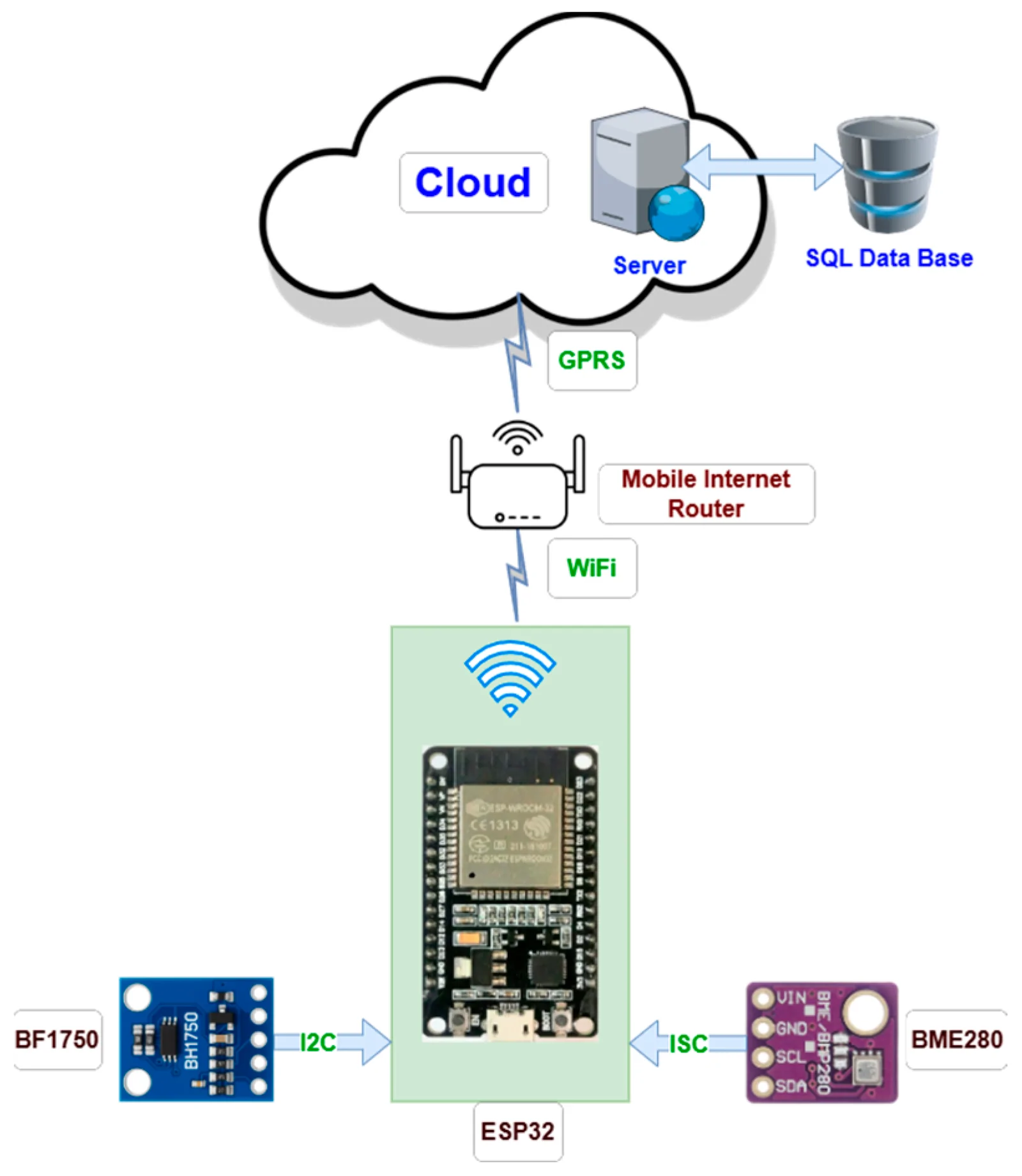
Automatic Weather Station schematics.

Each AWS (Automatic Weather Station, Figure 1) node is composed of two sensing elements connected via I2C to an ESP32 microcontroller (Espressif Systems, Shanghai, China): a BH1750 digital ambient light sensor and a BME280 environmental sensor. The BH1750 is a 16-bit light intensity sensor produced by ROHM Semiconductor [35] and works in high-resolution mode. Its raw register output is converted directly into irradiance in firmware using the physics-based model of the conversion framework of Michael et al. [30]:

G = E_raw × 0.0079 × (69/32) × 1.13 × 0.85 = E_raw × 0.01635 [W/m^2^], (1)

In this expression, *E_raw* is the raw BH1750 register value, the factor 0.0079 × (69/32) is the sensor-specific lux conversion including the MTreg correction, 1.13 is an empirically determined atmospheric transmittance factor and 0.85 accounts for the attenuation of the protective plastic dome. The conversion happens in firmware, so the database contains W/m^2^ values directly. The maximum values obtained (990 W/m^2^ for the mountain site and 1069 W/m^2^ for the rural site) are within the physically expected range.

The microcontroller is an ESP32-D0WD-V3 (silicon revision 3.01) on an ESP32 DevKit V1 board, dual core at 240 MHz with 4 MB of flash and 520 kB of SRAM, of which 331 kB is free at run time. These figures define the resource envelope within which the on-node inference of Section 5.4 must fit and are therefore stated explicitly.

The BME280 (Bosch Sensortec [36]) measures atmospheric pressure (300–1100 hPa, ±1 hPa), air temperature (−40 to +85 °C, ±1 °C), and relative humidity (0–100% RH, ±3%). The data is sent every 10 min via Wi-Fi to a remote MySQL database by the ESP32 [37]. Each record has timestamp, irradiance (W/m^2^), pressure (hPa), temperature (°C), humidity (%), wind speed and direction, and photovoltaic panel output voltage.

### 3.2. Network Topology, Communication Protocol and Link Reliability

The deployment consists of two physically built nodes operated in three successive site configurations. AWS1 was installed on the mountain (AWS1-M) from August 2024 to October 2025 and, after refurbishment, redeployed to the urban site (AWS1-U) from March to May 2026; AWS2 operated at the rural site (AWS2-R) from May to August 2024. The three station labels therefore denote three site deployments and not three concurrent nodes.

The topology is a star. Each node is associated with a local IEEE 802.11 b/g/n access point and reaches the server over the public internet; there is no node-to-node traffic and no mesh formation, so the system is not a multi-hop wireless sensor network and is not presented as one. What distinguishes it from isolated dataloggers is common firmware, schema and time base, and remote addressability.

Each node acquires both sensors over I2C and publishes one record of approximately 180 bytes to the server. The nominal acquisition period is ten minutes; the interval realized in the field, measured as the median difference between consecutive stored timestamps, is 619 s at AWS2-R and 626 s at AWS1, so the expected yield is 139.6 records per day rather than 144, and that value is the denominator used below. Timestamps are written by the server on insertion, so they carry the transmission latency.

The three sites present very different radio environments, open ground at the rural site, a terrain-obstructed path at the edge of coverage on the mountain, and dense multipath with heavy co-channel occupancy in the city center. Link performance was derived from the ingestion log rather than asserted. Table 1 reports, per deployment, the calendar days in the window, the operational days (days carrying at least ten records, excluding days lost to complete outage), the median daily yield, and the delivery ratio computed as mean daily yield on operational days divided by 139.6.

The ordering follows the radio environment. The open rural site delivers 96.8% of expected records; the mountain site delivers 83.5%—the node remaining functional but the obstructed uplink costing roughly one record in six; the urban site delivers 58.2%—the largest single degradation of the campaign. This is worth stating plainly, because the urban site is also the one whose irradiance signal is most attenuated: the deployment position penalizes the optical and the radio channel simultaneously, so any conclusion from that station rests on a record that is both weak and sparse. Received signal strength and retry counts are not logged by the firmware, so the delivery ratio is the only radio-layer metric that is reconstructable from the archive; instrumenting the nodes to log it is a planned firmware change.

The star topology scales linearly with node count if each node has an access point with a usable backhaul, and it is this criterion, not cost or power, that limited site selection. The mountain and rural positions were chosen partly because such an uplink existed, which excludes most of the topographically interesting locations in the Ovčar–Kablar gorge. A low-power, wide-area uplink removes that constraint and the 180-byte record fits within two LoRaWAN frames at DR3 in EU868. Migration is the planned path for network extension; no LoRa deployment is claimed.

### 3.3. Server and Database Architecture

The server tier is a single machine maintained in-house, running MySQL with an ingestion endpoint that validates the record and performs the insert. Measurements are stored in one wide table keyed by station identifier and timestamp. At two records per ten minutes the ingest rate is below 0.01 writes per second and the accumulated volume after two years is 47,125 rows, several orders of magnitude below the point at which the columnar compression and retention policies of a dedicated time-series engine would confer any advantage, while the relational engine provides mature access control and trivial backup. The choice is deliberately conservative and appropriate at this scale.

The limits of the arrangement should be stated as plainly as its merits. The database is not the binding constraint, since a single instance would sustain the insert load of several thousand nodes at this cadence; the binding constraints are the absence of replication, which makes the server a single point of failure, and the absence of scheduled backups. The nodes do not buffer: the archive contains no record whose timestamp precedes its predecessor by insertion order, so a record that fails to transmit is lost rather than replayed, which is also why the delivery ratios of Table 1 represent permanent data loss. A message broker with persistent sessions in front of a replicated instance, and node-side buffering in non-volatile storage, are the recommended evolution.

### 3.4. Security of the Acquisition Chain

Because every model trained on this record depends on the record not having been altered, the protections in place are stated without enhancement. Transport is plain HTTP without TLS. The node identifies itself with a station identifier carried in the record and there is no authentication beyond it, so the endpoint accepts any well-formed record presenting a valid identifier. Timestamps are assigned server-side, which prevents a client from backdating a record but does not authenticate its origin. Backups are not scheduled.

The resulting exposure is real and is not minimized here: traffic can be intercepted or modified in transit, and synthetic records can be injected by anyone who learns an endpoint and an identifier. What limits the practical risk is that the deployment is small, unadvertised and carries no personal data, and that the archive is internally consistent—the diurnal structure, the panel-to-irradiance relation and the quality-control retention rates show no discontinuity suggesting tampering. This is an argument for the integrity of the present dataset, not for the adequacy of the design. TLS with certificate verification, per-device tokens held in encrypted non-volatile storage, insert-only database privileges and scheduled off-host backups are the required hardening and are the first item of planned work on the platform.

### 3.5. Energy Harvesting and Autonomous Operation

Each node is powered by the resource it measures: a photovoltaic panel charges a battery through a charge controller, with no mains connection at any site. The panel output voltage is telemetered with every record, giving a continuous and independent measurement of harvesting conditions (Table 2). Its identity is explicit: the correlation between panel voltage and concurrent irradiance is 0.967 over the mountain deployment against 0.22 for wind speed, and the voltage is identically zero at night. It measures source availability, not battery state of charge.

**Figure 2:**
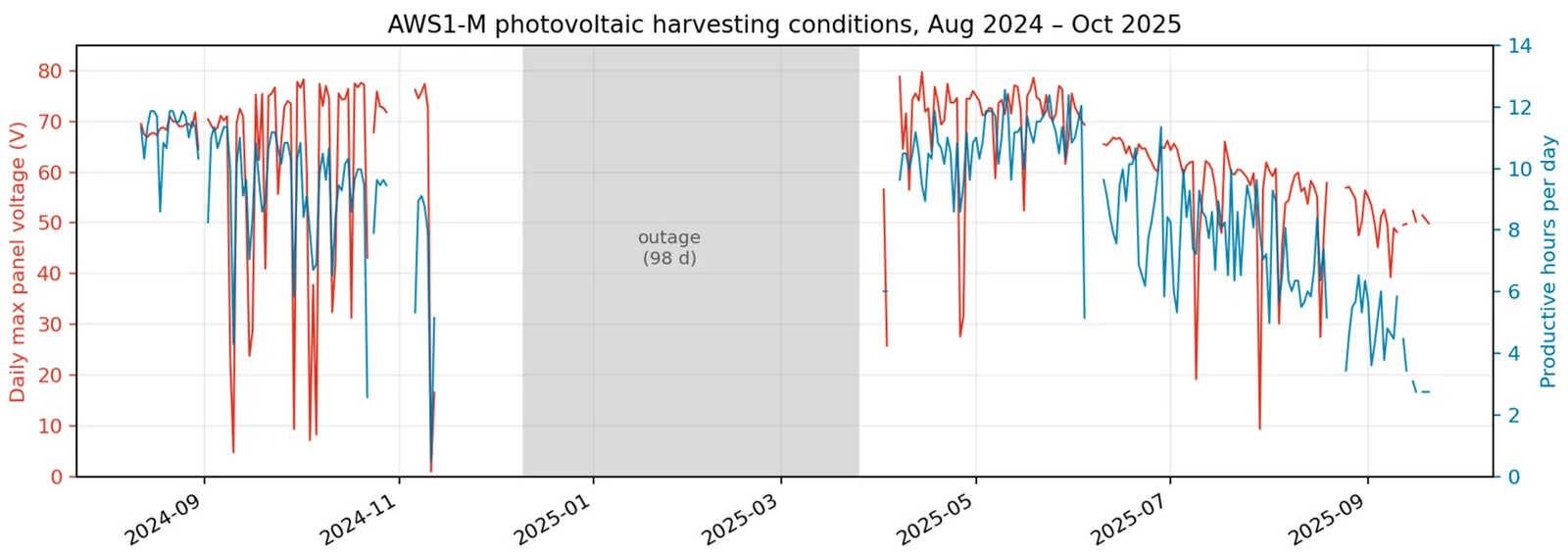
Daily maximum photovoltaic panel voltage and productive hours per day at AWS1-M, August 2024–October 2025, with the 98-day winter outage shaded.

Productive hours fall from about eleven per day in summer to 3.6 in November and 1.4 in December, while the daily voltage integral in November is roughly 0.8 of the April–May value. The two decline at very different rates because the panel still reaches a high midday output in winter, the 95th percentile of midday voltage is 63 V in December 2024 against 74 V in May 2025, while the window over which it produces anything shortens fivefold. Peak irradiance is therefore not the constraint in this environment; day length is. The longest run of consecutive days below 30% of the summer median integral was three days, which is the condition a battery sized for this site must absorb (Figure 2).

No energy-aware mechanism is implemented in the firmware. The acquisition interval is 619 s at night and 619 s at midday, and is unchanged between records taken while the panel produced no output and records taken at full output, so neither nocturnal duty-cycle reduction nor an adaptive cadence is present. No record is separated from its predecessor by less than 60 s, so transmissions are not batched. The node wakes, associates and publishes once every ten minutes throughout the twenty-four-hour cycle. This is a genuine inefficiency: 48% of the AWS1 archive is removed by the nocturnal stage of the quality control, so roughly half the daily radio activations produce records that no analysis uses. Suppressing or batching them or keying the cadence to the already-telemetered panel voltage, would remove that half at no cost to any result reported here. Neither was implemented, because the nodes were commissioned for continuity of measurement rather than minimum energy, and the harvesting budget proved sufficient without them.

The limitation of this account is that no direct electrical measurement was performed: supply current in the sleep, acquisition and transmission phases was not instrumented, so per-cycle charge, daily consumption and battery-sizing margin cannot be reported, and neither can a quantitative Wi-Fi against LoRaWAN comparison. A shunt-based characterization of the three phases is the immediate next step.

### 3.6. Measurement Sites

**Figure 3:**
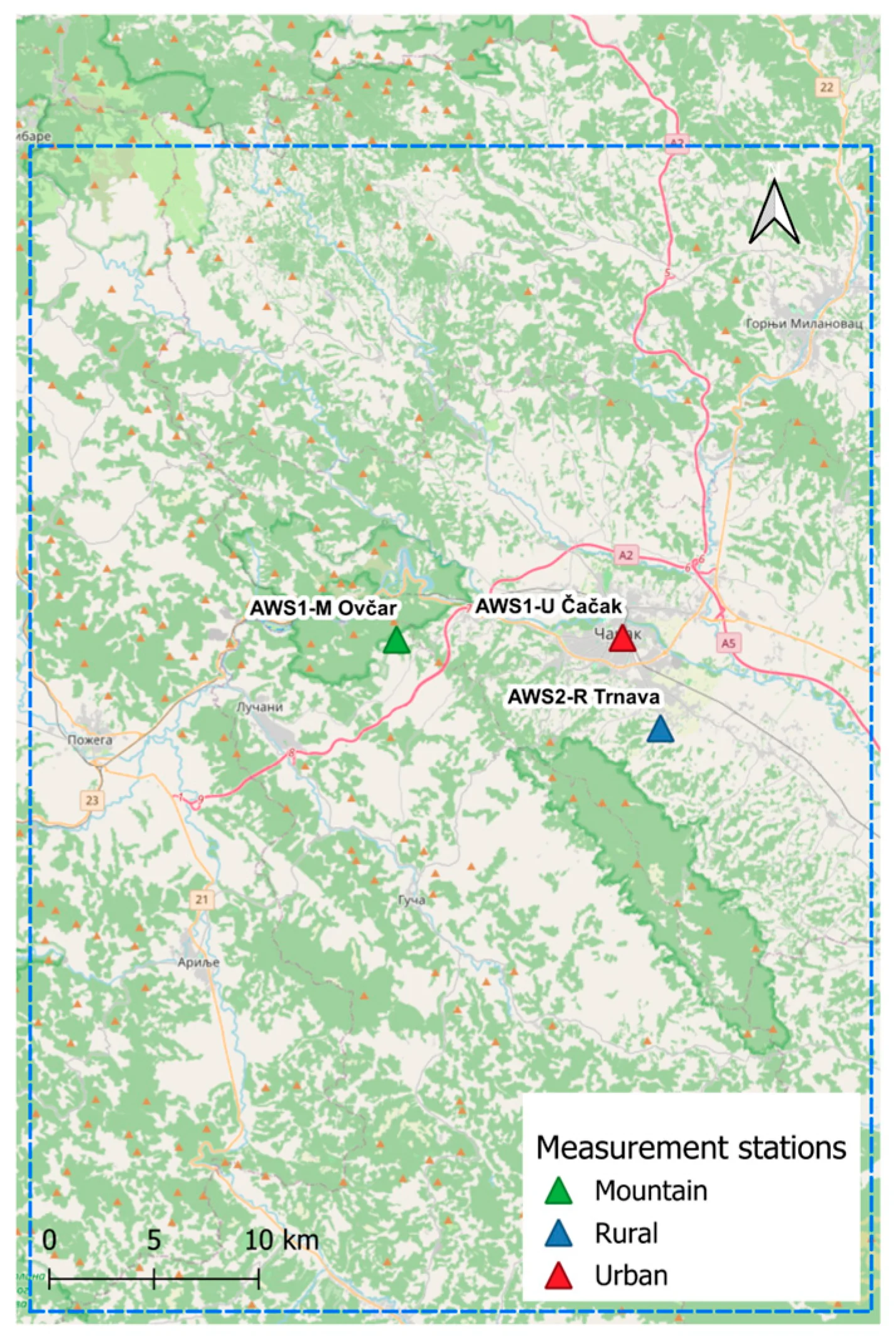
Geographic distribution of measurement sites in the Čačak region, Serbia. All three sites fall within a single NASA POWER 0.5° × 0.5° grid cell (dashed boundary). Latitude alignment of the sites (~43.85–43.89° N) removes geographic latitude as a confounding variable in inter-site comparisons.

Three sites were selected within a 25 km radius to represent the land-use categories of mountain, rural and urban areas. The station characteristics are summarized in Table 3 and their geographical distribution in Figure 3.

The three deployments never overlapped in time. Every inter-site difference reported in Section 6 therefore contains an inseparable contribution from inter-annual and seasonal meteorological variability, and none can be attributed to site geometry alone.

**Figure 4:**
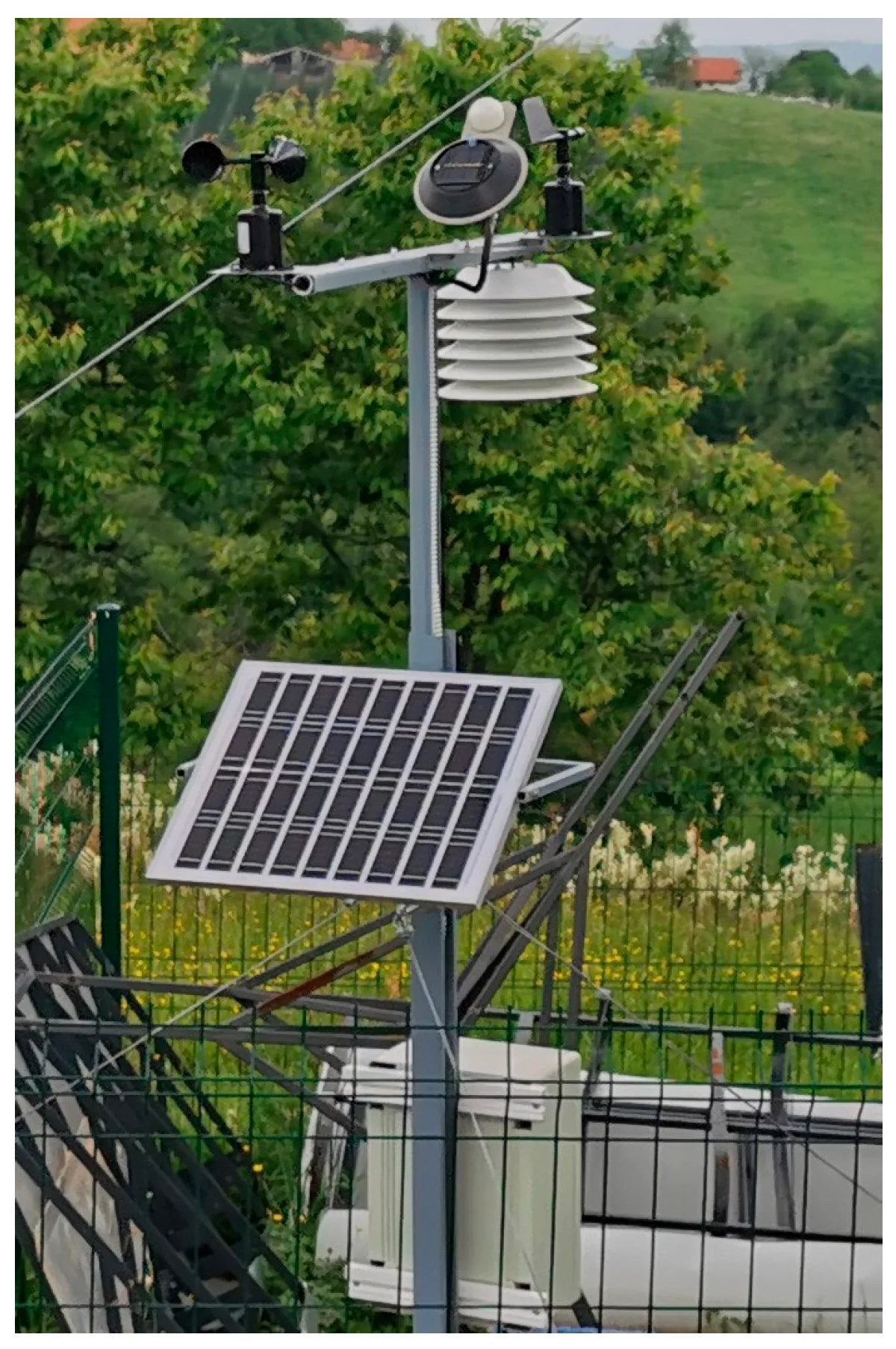
Photo of the AWS at the Trnava location.

AWS1-M was placed on the southern slopes of the Ovčar mountain in the village of Dučalovići, Ovčar-Kablar gorge. The terrain there is tough: the ridge line blocks a large part of the eastern horizon, the slope is directly exposed to the west in the afternoon, and convective clouds develop frequently over the massif in summer. AWS1-U was subsequently relocated to the center of Čačak, where it is surrounded by multi-story buildings and mature deciduous trees. AWS2-R has been installed in the open agricultural land of the Trnava village and is the free lowland site, Figure 4.

Note that the three sites are in almost the same latitude (~43.85–43.89° N) and are mainly separated in longitude (~20.22° E to ~20.37° E). Therefore, latitude is not a confounding factor in comparisons. The remaining differences are elevation, terrain complexity, and land use. In addition, all three sites are in the same NASA POWER grid cell, so the satellite reference is the same for all three. This fact alone illustrates the need for local ground measurement wherever sub-grid variability is of interest.

### 3.7. Sensor Calibration, Measurement Accuracy, and Uncertainty

The BH1750 is a commercial ambient light sensor, not a meteorological instrument, so the use of it for irradiance estimation introduces several sources of systematic and random error that must be stated explicitly.

The biggest systematic effect is spectral mismatch. The photopic sensor response peaks around 560 nm and is close to human eye sensitivity. The solar spectrum is spread from about 280 to 4000 nm. Approximately half of the clear-sky irradiance is in the ultraviolet below 400 nm and in the near-infrared above 700 nm to which the sensor does not respond. The firmware model partly compensates for this with the empirical transmittance factor of 1.13, but the correction is constant and independent of solar zenith angle, turbidity or cloud optical depth, all of which affect the spectral composition of the incident radiation [30].

A second systematic contribution is provided by the dome attenuation factor of 0.85, applied uniformly in firmware. The real transmittance of the dome is a function of the angle of incidence, the surface soiling and the aging of the plastic. No cleaning or inspection of the domes was done during the campaign, so there is a drift component of unknown magnitude. This is most likely at the mountain station, which was operated unattended for long stretches.

The BH1750 has a measurement reproducibility of ±20% under standard conditions as specified by the manufacturer [35]. Considering the conversion uncertainty, the absolute accuracy of the derived irradiance is estimated to be ±25–30% under clear sky conditions, and is worse under diffuse conditions, where the spectrum deviates more from the calibration assumption. Similar values have been reported for other low-cost illuminance-based irradiance sensors [29,31].

The dome attenuation factor of 0.85 is applied uniformly in firmware while the real transmittance depends on angle of incidence, soiling and aging. No cleaning or inspection was carried out, and the mountain station was not visited during the deployment, so a drift component was expected.

The decline is real and not seasonal, as can be seen from Table 4: between the identical calendar months of August–October 2024 and August–October 2025, the mean quality-controlled daytime irradiance falls from 334.5 to 193.3 W/m^2^, a reduction of 42% over months representing the same position in the annual cycle. The decline is shared: a linear fit over months with more than 200 valid midday records gives −35.5 W/m^2^ per month for the irradiance channel (*p* = 0.0007) and −2.38 V per month for the panel (*p* = 0.010), about 35% and 25% in relative terms. The ratio between channels drifts only weakly, by +1.1 per month.

The random error is only caused by the digitization noise (±1 lux in high resolution mode, i.e., ±0.016 W/m^2^ before firmware scaling), the thermal sensitivity of the sensor (about ±3% over the range −10 °C to +50 °C) and the latency in the wireless transmission, which can displace the stored timestamps by fractions of a minute.

No independent calibration against a reference pyranometer was performed. This is the main weakness of the uncertainty characterization. The comparison against NASA POWER hourly data provides a partial alternative: RMSE values of 240.74 W/m^2^ (AWS1-M) and 212.69 W/m^2^ (AWS2-R) include the combined uncertainty of the sensor system and the NASA POWER product itself, for which an uncertainty of 15–20% is estimated for hourly values in this region [1]. Future work includes crosscalibration against certified pyranometer measurements from RHMZ [34].

### 3.8. Quality Control and Filtering of Data

Raw records retrieved from the MySQL database were subjected to a multi-step quality control procedure that was applied similarly at all stations (Table 5).

Stage 1, Nocturnal exclusion: The threshold for AWS1-M and AWS2-R was 5 W/m^2^, which eliminates readings taken during twilight and nighttime that contain sensor noise rather than solar input. For AWS1-U the threshold was set to 0 W/m^2^ (strict zero exclusion), because in that strongly attenuated environment a 5 W/m^2^ threshold would have removed most of the valid daytime data.

Stage 2, physical plausibility: Records beyond physically plausible limits were flagged and removed; irradiance above 1100 W/m^2^, air temperature outside −30 to +50 °C, relative humidity outside 0–100%, pressure outside 900–1100 hPa.

Stage 3, gap analysis: Time gaps longer than 60 min were identified and recorded. Measurements within known downtime (electronics failure, November 2025–March 2026) were excluded completely. Gaps are not interpolated. Missing periods are present in all analyses.

Stage 4, Fault elimination (sensor): The pressure sensor BME280 at AWS1 shows a systematic offset of about +45 hPa since end of March 2025, most probably after a sensor replacement without re-calibration. Therefore, pressure data from the period April 2025–May 2026 were excluded. No such fault was detected on the temperature and humidity from the same unit, and these were kept.

## 4. Machine Learning Models and Evaluation Metrics

The detailed architectural descriptions are given in Appendix A; this section keeps what is needed to read the results and the deployment analysis. Twenty-five model configurations were trained, drawn from six families.

XGBoost [9] is an ensemble of regression trees fitted with second-order gradient information, regularization of leaf weights, and tree pruning, wrapped in a multi-output regressor. The recurrent group comprises LSTM [10] and GRU [11] stacks of one, two and three layers with 64/32/16 hidden units. The convolutional group comprises three 1D CNN variants of increasing depth and three TCN stacks [17,18] based on dilated causal convolutions. Six hybrids combine a convolutional or temporal convolutional front end with a recurrent back end, and two LSTM-GRU stacks combine the two recurrent cell types. TCN-GNN encodes each 12-month window as a chain graph whose nodes are individual months, extracts per-node features with a two-level temporal convolutional network and propagates them with two graph convolutional layers [25], following spatio-temporal designs such as Graph WaveNet [26].

A plain feed-forward network with ReLU activations was added, since it is among the most widely used baselines for time-series. The architecture is a flattened 12-month input window followed by dense layers of 128, 64 and 32 units with ReLU activation and dropout of 0.2 after each, and a linear output of two units. It is trained under the same protocol as the other Keras models, so the comparison is not confounded by optimization settings. A second, deliberately smaller, fully connected variant (72-32-16-2, without dropout) was trained for on-node execution.

All Keras models terminate in a dropout layer of rate 0.2, a dense layer of 16 units with leaky-ReLU activation and a linear output of two units and were trained for 100 epochs with Adam at learning rate 0.01 and mean-squared-error loss. TCN-GNN was implemented in PyTorch Geometric 2.11 and trained for 100 epochs with Adam at 0.001 and smooth-L1 loss. Evaluation metrics are MAE, MAPE, MSE, RMSE, MSLE, RMSLE, R^2^ and adjusted R^2^, defined in Appendix A.

## 5. Reference Data and Satellite-Series Modeling

Hourly global horizontal irradiance (GHI) data from the NASA POWER (Prediction Of Worldwide Energy Resources) satellite [1] was obtained for the study coordinates via the NASA POWER API. All three AWS sites fall within the same 0.5° grid cell and therefore a single reference series can be used. The hourly series used for the matched-pair validation covers the period from May 2024 to December 2025 (7593 hourly values in daytime). The archive was then expanded, and daily data were available until 31 March 2026 at the time of the final analysis. Quantitative validation on matched hourly pairs was performed for AWS1-M and AWS2-R. The reference for the AWS1-U window (March–May 2026) was built from the archived March 2026 data and a model-based reconstruction of April and May 2026, as explained in Section 5.3.

### 5.1. Temporal Alignment Procedure

The two temporal resolutions must be reconciled before AWS ground data can be compared to NASA POWER. The AWS stations provide instantaneous irradiance measurements at 10 min intervals. The values from NASA POWER are hourly integrated, where the value is the average flux for the previous clock hour (e.g., the 10:00 value is for 09:00–10:00 UTC).

For the derivation of hourly mean values AWS 10 min records were aggregated to hourly means with a strict completeness rule, i.e., hourly, means were only calculated if at least three out of six possible 10 min records in that hour were valid (minimum 50% availability). Hours not following the rule were discarded, since incomplete averages of hours would introduce a systematic bias.

NASA POWER timestamps are in UTC. The AWS loggers are in local time (CET = UTC+1 in winter, CEST = UTC+2 in summer). AWS timestamps were converted to UTC prior to matching and daylight-saving transitions (last Sunday of March and October) were explicitly handled.

Hourly pairs were built after aggregation and conversion to UTC by an inner join on the timestamp index, and only pairs with both values above zero were kept. This yielded 3072 matched pairs for AWS1-M (Ovčar) and 1202 for AWS2-R (Trnava) for the periods August 2024–October 2025 and May–August 2024 respectively.

### 5.2. Regression Modeling of the Long-Term Satellite Series

The complete set of models was retrained on the long-term NASA POWER record for the Čačak grid cell to link the local measurements to the machine learning framework. The daily data for the point 43.89° N, 20.35° E were aggregated to monthly means for the period January 2001–March 2026, providing 303 complete monthly values of four parameters: the all-sky insolation clearness index (ASICI), the all-sky surface albedo (ASSA), and the all-sky and clear-sky surface shortwave downward irradiance (ASSSDI, CSSSDI). No missing values were present in this range, and no imputation was performed at any stage. The two irradiance variables were used as regression targets and ASICI and ASSA were used as predictors. We supplemented the feature set with sine and cosine transformations of the calendar month, a running time index and a numeric timestamp. All features and targets were min–max scaled. The train/test split was chronological (80/20, no shuffling) and the sequential architectures were given sliding windows of 12 consecutive months.

In total, 24 models were trained. XGBoost [9] is based on the gradient boosting family: an ensemble of regression trees fitted with second order gradient information, regularization of leaf weights and tree pruning, wrapped in a multi-output regressor so that both irradiance targets are predicted together. The recurrent group consists of stacks of LSTM [10] and GRU [11] units with 1, 2 and 3 layers (with 64/32/16 hidden units in the following layers), which can model temporal dependencies via gating mechanisms. The convolutional group includes three variants of 1D CNN with increasing depth and 3-width kernels, max-pooling and global average pooling. The temporal convolutional group consists of three TCN stacks [17,18], which are based on dilated causal convolutions and have an exponentially growing receptive field with depth. Six hybrids combine a convolutional or temporal convolutional front end with a recurrent back end (two depths each of CNN-LSTM [21], CNN-GRU, TCN-LSTM and TCN-GRU), and two stacks of LSTM-GRU combine the two recurrent cell types. The last layer of each Keras model was a dropout layer (rate 0.2) followed by a dense layer of 16 units with leaky-ReLU activation and a linear output of two units. The models were trained for 100 epochs with the Adam optimizer (learning rate 0.01) and a mean-squared-error loss.

The last model TCN-GNN is designed with graph-hybrid. Each 12-month input window is encoded as a chain graph with the nodes being the individual months, linked bidirectionally to their temporal neighbors. It first uses a two-level temporal convolutional network (with 16 and 32 channels and causal convolutions with dilation) to extract per-node features. The information is then propagated along the chain by two graph convolutional layers [25] (32→16 and 16→8 channels) following the approach of spatio-temporal graph models such as Graph WaveNet [26]. The two regression outputs are obtained by global mean pooling and a linear layer. The model was implemented using PyTorch with PyTorch Geometric and trained for 100 epochs with Adam (learning rate 0.001), smooth-L1 loss and checkpointing based on the test loss.

The performance was evaluated on the held-out final fifth of the record using the following metrics: MAE, MAPE, MSE, RMSE, MSLE, RMSLE, R^2^ and adjusted R^2^. The full metric table for all 25 configurations is given in Appendix B, Table A1; Table 6 retains the per-family summary needed to follow the argument.

**Figure 5:**
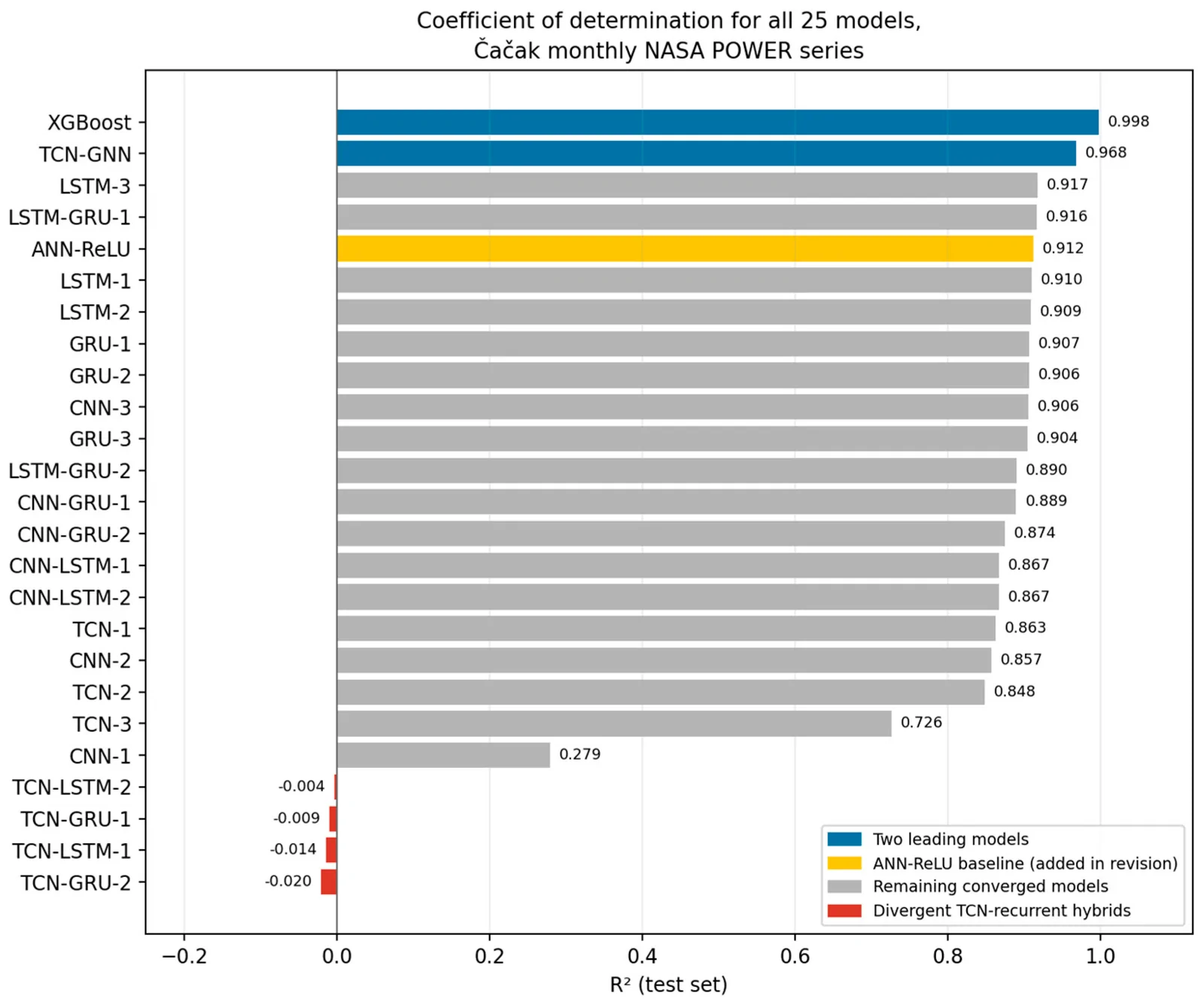
Coefficient of determination (test set) for all 25 models trained on the Čačak monthly NASA POWER series.

**Figure 6:**
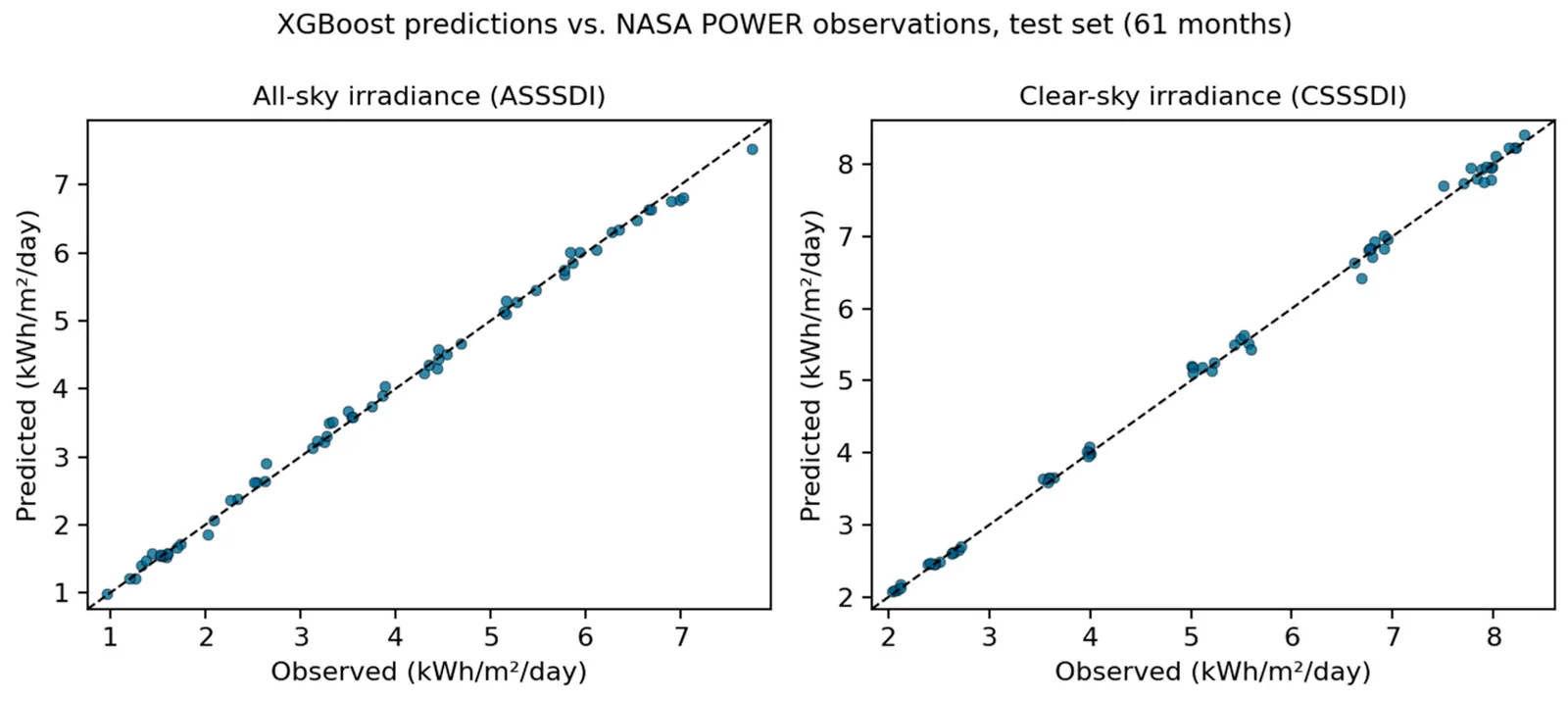
XGBoost predictions against NASA POWER observations on the 61-month test set, for the all-sky (**left**) and clear-sky (**right**) irradiance targets. The dashed line marks perfect agreement.

As can be seen from Figure 5, XGBoost is the best model (R^2^ = 0.998, RMSE = 0.093 kWh/m^2^/day), as expected for a moderate-size tabular dataset with strong seasonal structure. The best neural architecture is TCN-GNN (R^2^ = 0.968), followed by the plain LSTM stacks (R^2^ ≈ 0.91–0.92) and the CNN-GRU and CNN-LSTM hybrids (R^2^ ≈ 0.87–0.89). The added ANN-ReLU baseline reaches R^2^ = 0.912, placing it fifth overall, between LSTM-GRU-1 and LSTM-1. A plain fully connected network matches three-layer recurrent stacks indicating that sequential modeling adds little over ordinary regression on calendar and satellite features for this dataset, while gradient boosting remains clearly ahead. The single-layer CNN fails to capture the seasonal cycle (R^2^ = 0.28) and the four TCN-recurrent hybrids diverge under the common training protocol. We present the fit of the leading model on the test period in Figure 6.

### 5.3. Reference Reconstruction for the 2026 Gap

The NASA POWER archive for the time of analysis stopped on 31 March 2026, and therefore no concurrent satellite reference was available for April and May 2026, two out of the three months of AWS1-U operation. The models presented in Section 5.2 use concurrent satellite parameters as inputs and cannot be applied to months for which no satellite data is available. For extrapolation a separate seasonal XGBoost model was fit with only calendar features (monthly sine and cosine terms and a running month index), allowing for true forward prediction. For all-sky irradiance this model achieves R^2^ = 0.928 (RMSE = 0.506 kWh/m^2^/day) on the held-out most recent 20% of the record, and R^2^ = 0.998 for clear-sky irradiance.

**Figure 7:**
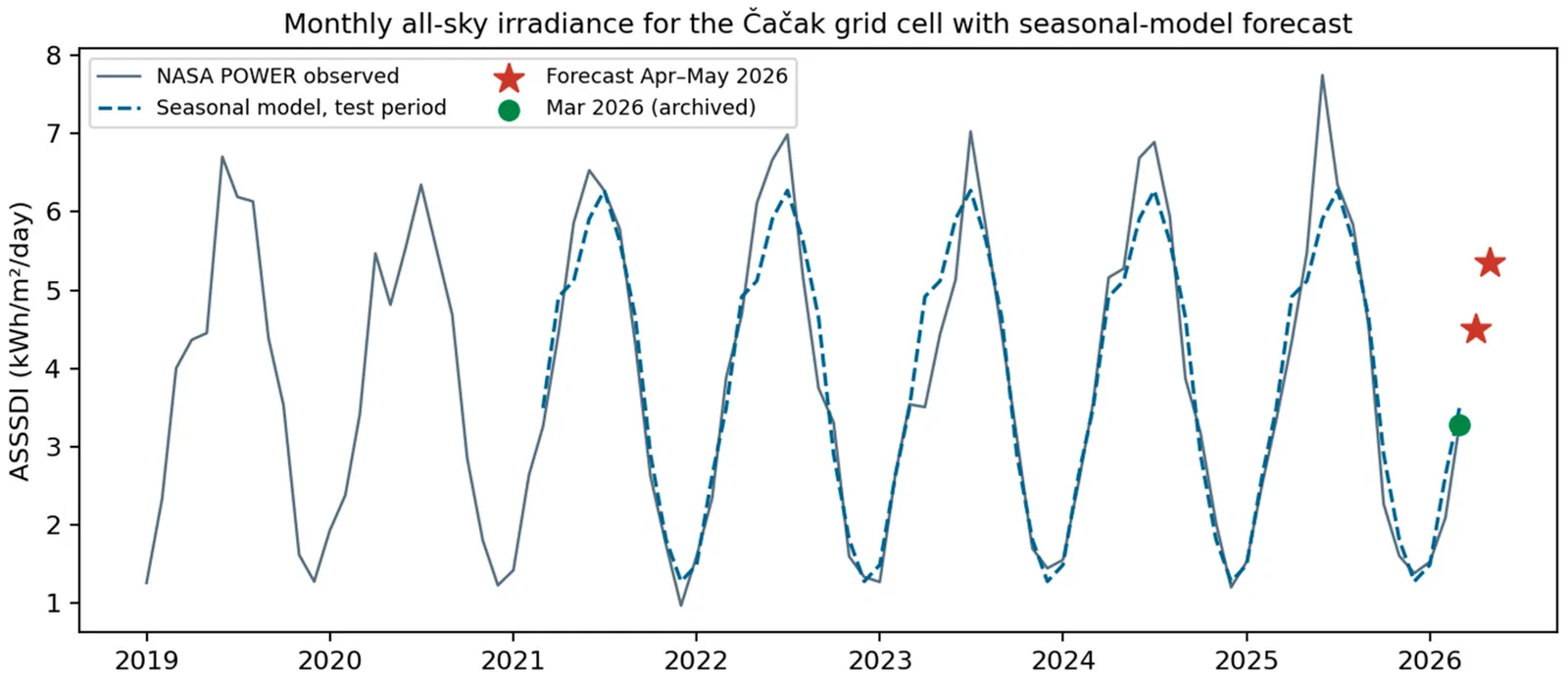
Monthly all-sky irradiance for the Čačak grid cell (2019–2026). The dashed line shows the seasonal model over the chronological test period; the green marker is the archived March 2026 value, and the red stars are the model’s April–May 2026 forecasts.

The model predicts 4.50 and 5.36 kWh/m^2^/day for April and May 2026 respectively. This converts through the astronomical day length at the station latitude to mean daytime irradiances of about 340 and 368 W/m^2^. For AWS1-U the window averages 329.6 W/m^2^ (Table 7, Figure 7) with the archived March 2026 value (3.28 kWh/m^2^/day, about 281 W/m^2^). The previous-year proxy (March–May 2025) yields 330.3 W/m^2^, a difference of 0.2%, and hence the two approaches corroborate each other. This reconstructed value is adopted as the AWS1-U reference for the rest of this section.

### 5.4. Edge Deployment of the Regression Models

The models above were trained and evaluated on a workstation, which says nothing about whether the nodes that produced the ground data could execute any of them. This section answers that question by direct measurements on hardware (Table 8). The purpose is not to accelerate the monthly satellite regression, which has no real-time requirement, but to establish what class of model an ESP32 node can host so that it can correct its own bias and detect its own faults without a round trip to the server.

Two conversion paths were used, both without a third-party inference runtime. The gradient-boosted ensemble was compiled to straight-line C in which each tree is a nest of comparisons on the feature vector and the prediction is the sum of leaf values; a depth-3 ensemble of 40 trees per target was retrained for this purpose. The fully connected network was quantized to INT8 with per-tensor symmetric scaling and executed by a hand-written kernel. Correctness was verified before timing: the on-device outputs agree with the workstation reference to eight decimal places for both models. Latency was measured over 1000 repetitions at 240 MHz with a varying input, so that the compiler could not hoist the computation out of the loop, and a control loop measuring only the timing overhead (0.601 µs) was subtracted.

Three points follow. First, both models fit the node with room to spare; together they occupy 14.2 kB of the 4 MB flash and 504 B of the 520 kB SRAM, and the 138 µs worst case is 0.00002% of the 619 s acquisition period. On-node inference is therefore not constrained by the hardware at this cadence. Second, the smaller model is the slower one. The network occupies a quarter of the flash of the tree ensemble yet takes three times as long, because a tree visits at most three comparisons per tree regardless of input while the network performs 2848 multiply–accumulate operations every time. What determines edge feasibility is the shape of the computation, not the parameter count. Third, the graph hybrid does not convert at all: graph convolution requires sparse adjacency operations and dynamic indexing for which no microcontroller runtime provides an implementation, so it is cloud-only by construction rather than by resource budget.

Energy places this in context, with the forewarning that it is estimated and not measured. At the datasheet supply current of approximately 40 mA at 240 MHz and 3.3 V, one inference costs about 6 µJ for the tree ensemble and 18 µJ for the network. A single Wi-Fi association and transmission, at a datasheet transmit current of about 200 mA over a few hundred milliseconds, costs on the order of 200 mJ—four orders of magnitude more. Local inference is therefore energetically free relative to the transmission the node already performs every ten minutes, which is what makes the transmission-gating discussed in Section 3.5 worth pursuing.

**Hierarchical Edge–Cloud Intelligence Pipeline:**

These empirical measurements define a distinct logical partitioning between edge execution and cloud infrastructure. At the edge tier, the low-power ESP32 microcontroller hosts lightweight models—specifically the depth-limited XGBoost ensemble (46.3 µs latency, 11.1 kB flash, 24 B RAM) and the INT8 quantized neural network (138.1 µs latency, 3.0 kB flash, 480 B RAM). Because on-node inference consumes negligible energy (~6–18 µJ) compared to the ~200 mJ required for a single Wi-Fi transmission, local inference operates inline prior to radio uplink. The primary responsibilities of this edge tier are:

1. *Real-time bias correction:* Dynamically adjusting raw illuminance-to-irradiance readings against known terrain and atmospheric transfer functions prior to storage or transmission.
2. *On-device anomaly and fault detection:* Evaluating prediction residuals against real-time measurements. Significant residual spikes indicate transient physical obstruction; whereas, persistent unidirectional drift flags sensor fouling, dome degradation, or calibration shift.
3. *Transmission gating:* Suppressing redundant or corrupted telemetry when harvesting conditions are depleted or when the sensor is confirmed faulty, directly conserving battery reserves.

Conversely, the **cloud tier** retains high-capacity sequence and graph models, notably TCN-GNN (R^2^ = 0.968) and multi-layer recurrent stacks (R^2^ approx. 0.91). These architectures require dynamic graph convolutions, sparse matrix operations, or substantial multi-step sequence buffers that cannot execute under the static memory constraints of the ESP32. The cloud infrastructure serves three roles:

1. *Periodic model retraining and recalibration:* Ingesting aggregated ground data across multiple nodes and new satellite releases to update model parameters and seasonal feature weights.
2. *Parameter downlink:* Compiling retrained decision trees into compact C structures or quantizing updated neural weights for periodic over-the-air (OTA) updates to the edge nodes.
3. *Macro-scale irradiance modeling and gap-filling:* Running multi-site cross-comparisons and forward satellite-proxy reconstructions across extended regional domains.

The scope of these measurements should be read narrowly. They were obtained on a single hardware revision at a fixed clock and ambient temperature; latency and energy will vary with clock scaling and regulator temperature. The corrector was validated off-line, so its closed-loop behavior, where its own gating decisions alter the record is later retrained on, has not been evaluated in the field. Also, it reduces bias against satellite reference, which is not the same as establishing traceable absolute accuracy.

## 6. Results and Discussion

### 6.1. Descriptive Statistics

**Figure 8:**
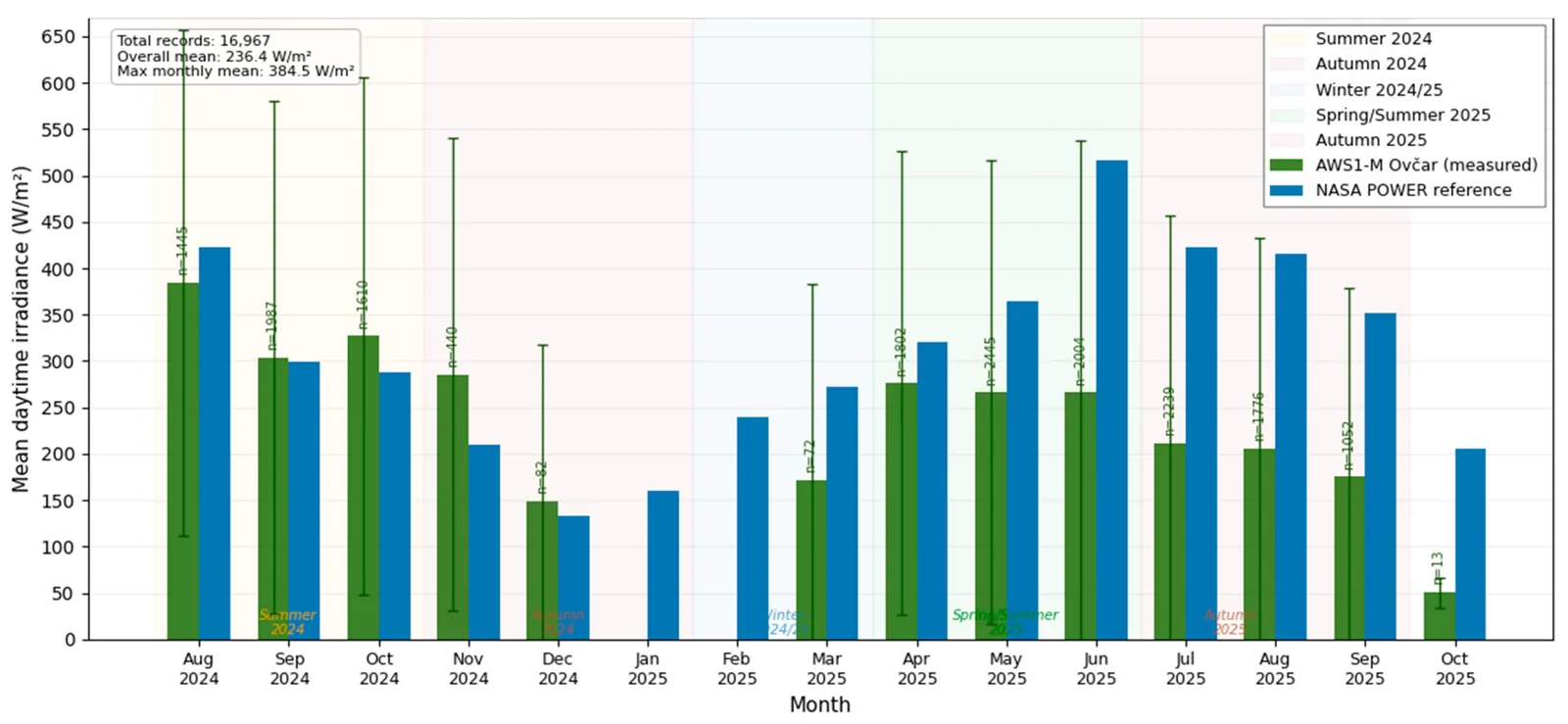
Monthly mean daytime solar irradiance at AWS1-M Ovčar (mountain site), August 2024–October 2025. Values are means of all quality-controlled daytime records (irradiance > 5 W/m^2^) within each calendar month.

Table 9 presents irradiance statistics for all sites combined with the NASA POWER reference. The highest mean (351.5 W/m^2^) was observed at the Trnava rural site, approximately 7% higher than the NASA POWER regional average of 329.5 W/m^2^, which is representative of an installation in an unobstructed open-field setting and appropriate sensor orientation. The mountain Ovčar site averaged 267.7 W/m^2^ or about 81% of reference; the shortfall is systematic and attributed to topographic shading. The urban Čačak site is a special case with a mean of 19.6 W/m^2^ and a maximum of 78 W/m^2^, or about 6% of the reference for the equivalent spring window, because of the surrounding buildings and tree canopy at the deployment position. Monthly mean daytime solar irradiance at AWS1-M Ovčar (mountain site) from August 2024 to October 2025 are given in Figure 8.

Because of the response loss documented in Section 3.7, the mountain record is reported over two intervals. The full-period value is retained so that the campaign statistics remain comparable with the other sites and with the first version of this work; the stable-period value covers August 2024 to May 2025, before the decline becomes significant.

The distinction matters for the interpretation. Over the full period the mountain site falls 61.6 W/m^2^ below the satellite reference; over the stable period the shortfall is 27.5 W/m^2^, or 92% of reference. Roughly half of the deficit previously attributed in its entirety to topographic shading is instrumental in origin.

### 6.2. Statistical Validation Against NASA POWER

Table 10 shows the validation metrics against NASA POWER hourly reference for AWS1-M and AWS2-R. Trnava showed the higher correlation (R^2^ = 0.567, RMSE = 212.69 W/m^2^) which supports its use as the representative ground reference for the region. Ovčar is similarly correlated (R^2^ = 0.489, RMSE = 240.74 W/m^2^) but has a much larger negative bias (MBE = −138.89 W/m^2^), consistent with the combined effect of ridge shading and convective cloud over the massif.

**Figure 9:**
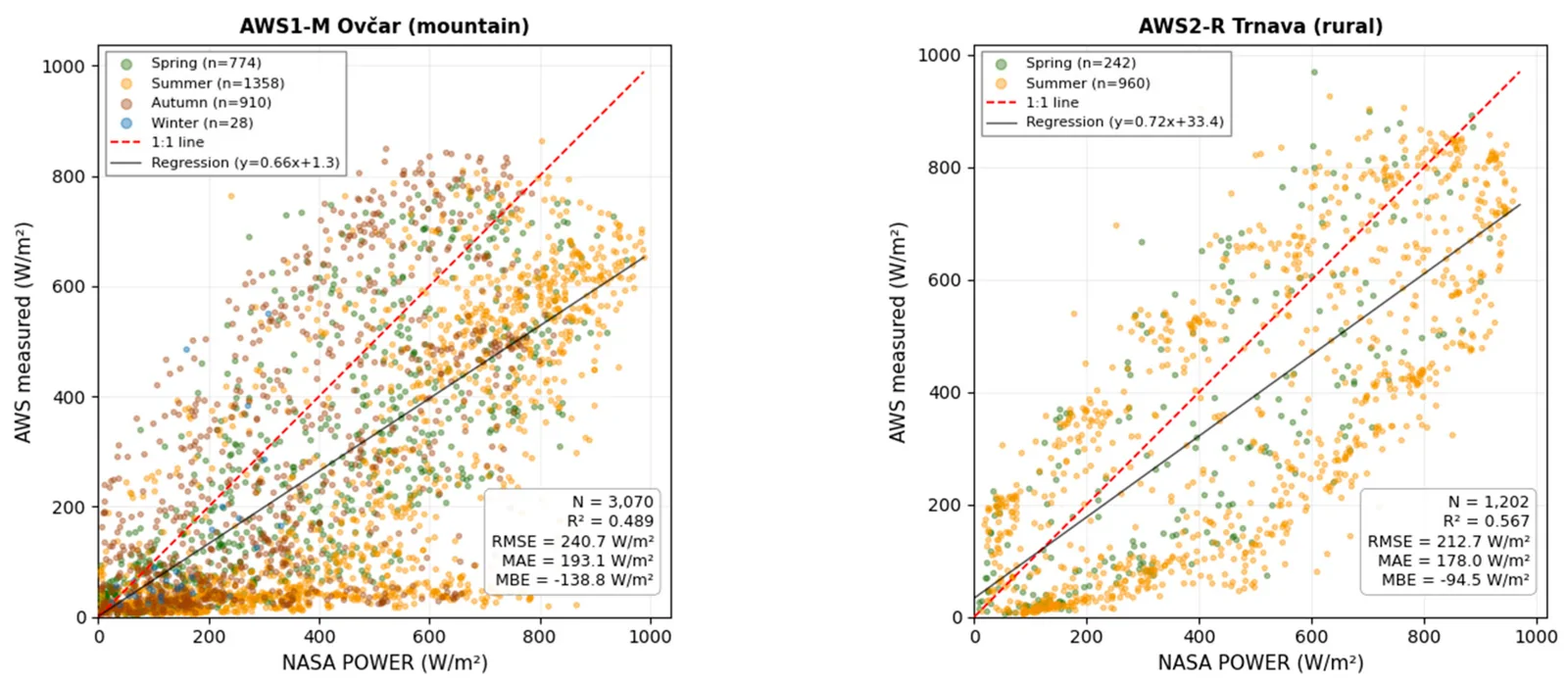
Scatter plots of hourly irradiance: AWS ground measurements vs. NASA POWER reference. (**Left**): AWS1-M Ovčar (N = 3072, R^2^ = 0.489, MBE= −138.9 W/m^2^). (**Right**): AWS2-R Trnava (N = 1202, R^2^ = 0.567, MBE= −94.5 W/m^2^). Dashed line indicates perfect agreement (1:1).

The seasonal decomposition of Ovčar is quite stable. The bias is lowest in spring (MBE = −105.21 W/m^2^, R2 = 0.592) and highest in summer (MBE = −218.10 W/m^2^, R^2^ = 0.566) when convective cloud development over the massif is most intense and biases the measured values low compared to the spatially averaged NASA POWER estimate [1]. The autumn values (R^2^ = 0.451, MBE = −52.84 W/m^2^) correspond to more stable conditions with little convection, as can be seen from Figure 9.

### 6.3. Diurnal Irradiance Patterns

**Figure 10:**
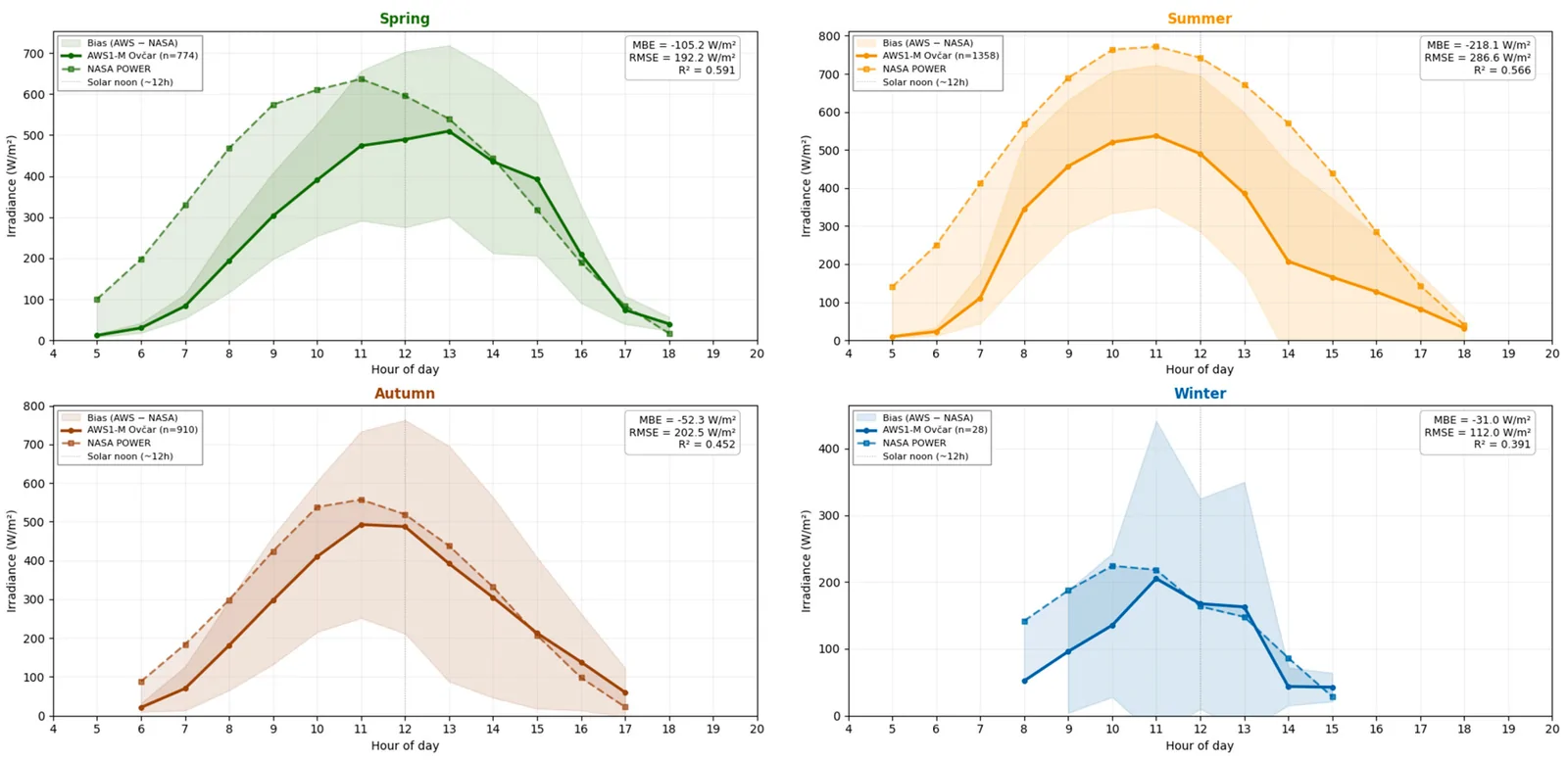
Mean diurnal solar irradiance profiles by season at AWS1-M Ovčar (solid lines) compared to the NASA POWER reference (dashed lines). Shaded areas indicate the bias between measurements and reference. The asymmetric morning/afternoon pattern reflects eastern horizon obstruction and western direct exposure characteristic of the mountain slope installation.

The mean diurnal profiles show distinct site signatures (Figure 10). The profile on Ovčar is asymmetric: in the morning the measurements are lower than NASA POWER because of the blocking by the eastern ridge of the low sun, and they are higher than the reference in the spring and autumn afternoons because of the direct western exposure of the slope. The largest negative hourly biases are −301 W/m^2^ in summer mornings (7 h) and −274 W/m^2^ in spring (8 h). In spring afternoons, the bias becomes positive (+75 W/m^2^ at 15 h). These values are combined to determine an east-facing slope installation.

Trnava is very close and symmetrical to NASA POWER curve with mean peaks about 600 W/m^2^ in spring and 750 W/m^2^ in summer. It is the nearest of the available sites to an unobstructed horizontal datum.

**Figure 11:**
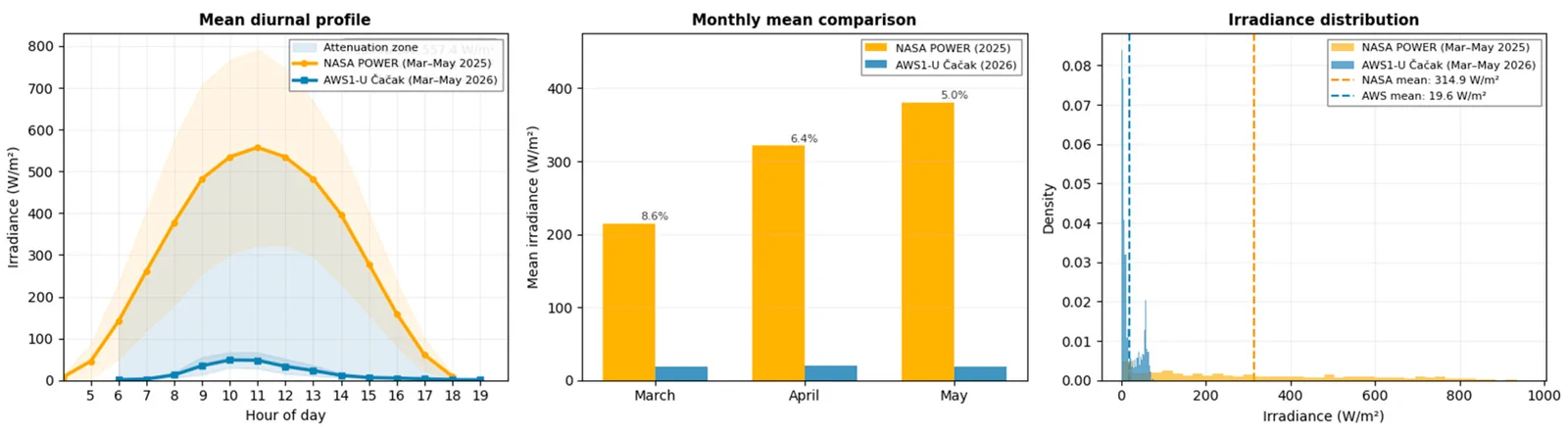
Mean diurnal solar irradiance profiles for the urban AWS1-U Čačak site (March–May 2026, solid line) compared to the NASA POWER reference (dashed line). The ~12:1 attenuation ratio indicates severe horizon obstruction by surrounding buildings and vegetation at the deployment location.

The urban site behaves quite differently (Figure 11). Its diurnal profile is flattened and irregular, with a peak of about 48 W/m^2^ between 10 and 11 h, compared with a reference peak of about 557 W/m^2^, i.e., an attenuation of roughly 12:1. At such a ratio, the sensor is effectively measuring the deployment microenvironment; i.e., building shadow and canopy, rather than the open-sky resource.

### 6.4. Implications for Solar Irradiance Prediction

The dataset provides some observations that can be directly used in the machine learning framework. The simplest one is that NASA POWER gives the same values for all three sites, but the sites behave very differently. A coarse resolution product is valuable for regional assessment but is unable to assess the solar resource at the micro-scale. Ground IoT networks, even those constructed from low-cost components, capture exactly the sub-grid variability that the satellite grid averages out.

The systematic negative bias at the mountain site has a practical implication: models trained only on satellite data will overestimate the resource, and installations sized on such estimates will underproduce in topographically complex locations. Because that bias is site-specific, persistent and known in advance, it is the case in which a small local model is preferable to a large remote one. The Trnava record, with 7248 quality-controlled measurements over the key summer months, is the most reliable ground reference produced in this campaign and is recommended as the primary dataset for model training and testing in the Čačak area.

The urban record cannot be used for open-sky irradiance estimation, but it is not useless: it shows concretely the fragmentation of the urban irradiance field by building geometry and vegetation. Models that include descriptors of obstruction such as sky view factor or height to width ratios of buildings [32] seem to be the realistic way towards useful predictions in such environments.

### 6.5. Urban Site Limitations and Representativeness

The attenuation degree at AWS1-U (the mean of 19.6 W/m^2^ and the maximum of 78 W/m^2^, about 6% of the reference) makes one wonder if these values describe the site or a wrong installation.

The evidence indicates the site. The hardware and firmware are the same as the mountain node whose irradiance ranged up to 990 W/m^2^, so the sensor system can measure high irradiance. The diurnal curve of AWS1-U is strongly attenuated but retains a bell shape with a midday peak around 10–11 h, i.e., the behavior of a working sensor following the sun. The maximum of 78 W/m^2^ also corresponds to partially obstructed clear sky conditions, and not the near zero output of a defective sensor.

Satellite imagery of the deployment site shows multi-story buildings to the east and south and mature deciduous trees to the west, together leaving only a narrow portion of sky visible. The sky view factor (SVF) given as the fraction of the sky hemisphere visible from the sensor [32] is well below 0.5 based on the surrounding morphology, while values at the open Trnava site reach almost 1.0. SVF is consistently reported in the literature as the dominant control on irradiance reduction in urban canyons with reductions of 30–90% for SVF < 0.5 [32].

This adds a seasonal mechanism to the geometric obstruction over the measurement window. The attenuation varies over the deployment period, because the western side of the site is shaded by deciduous trees, whose canopy is bare in March and fully developed by May. In March part of the direct beam still passes through the leafless crowns, in late spring very little of the canopy transmits. This is contrary to the open-sky seasonal cycle: the reference rises from 281 W/m^2^ in March to 368 W/m^2^ in May, but the effective transmittance of the micro-location drops and the relative gap between sensor and reference widens through spring although the sensor itself behaves consistently. The higher solar elevation during these months does not help, since the dominant obstruction is overhead (the canopy) and not just lateral (the buildings).

The strong influence of the tree crowns on the radiation reaching the ground is well known. The transmissivity of solar radiation through the crowns of single urban trees was measured by Konarska et al. [38], who found that a leafy crown transmits only a small fraction of incoming radiation, while the same crown without leaves in winter transmits several times more. Similar seasonal differences in the solar permeability of common urban tree species in Szeged were reported by Takács et al. [39]. Measurements in a tropical urban park [40] showed that transmissivity is also dependent on the species and on the share of diffuse radiation. Wu et al. [41] compared the shading effect of tree canopies and buildings in the urban environment by adopting the transmittance of solar radiation.

The geometry of the surrounding buildings follows the same direction. The shading of the canyon floor was highly dependent on the height-to-width ratio and the street orientation for urban canyons in a hot dry climate as shown by Bourbia and Awbi [42]. Rostami et al. [43] investigated the influence of the urban morphological parameters on solar potential, the energy consumption and the daylight autonomy of canyons and buildings. Experiments on a scaled street canyon [44] showed the redistribution of the solar radiation inside the canyon as a function of the geometry and the envelope materials. They support the interpretation that the low values at AWS1-U are the expected consequence of the deployment micro-location, where the canopy and the building shading act simultaneously.

Thus, the AWS1-U record is a description of one obstructed micro-location, not of the urban solar resource of Čačak in general. Two things happen. The dataset as a measurement cannot be used for city-scale resource assessment or direct comparison with open-sky products such as NASA POWER [1] without an explicit SVF correction. However, as an application dataset it is relevant for building-integrated photovoltaics, where the energy yield is precisely determined by the irradiance at partially shaded urban positions and not by open-sky values.

Without a co-located reference pyranometer and a sky camera to quantify the SVF, it cannot be decided whether the installation geometry accounts for the attenuation, or whether sensor orientation, dome soiling or local aerosol loading are also at play; this combination is a priority for future work. The AWS1-U dataset is kept for descriptive purposes until then, as an example of the difficulty of representative deployment in dense urban fabric.

The practical necessity of the hierarchical edge–cloud intelligence pipeline is demonstrated by the field failures observed during this campaign. The +45 hPa systematic pressure offset at AWS1 persisted for fourteen months before detection during post-deployment analysis, and the 42% optical response attenuation was identified only because a secondary radiometric panel channel happened to be telemetered. Had the depth-limited XGBoost model or INT8 network executed locally on the ESP32, an on-device residual thresholding filter would have flagged both faults within days of onset. Local fault classification enables immediate operational warnings and allows the node to autonomously gate transmission of invalid data, preventing database pollution and saving uplink energy.

### 6.6. Study Limitations

The main limitations of the study arise from the practical constraints of operating low-cost autonomous hardware in the field for a long duration.

**Continuity of measurement.** The AWS1 record contains two interruptions rather than the one reported in the first version. The longer is a 98-day outage from December 2024 to late March 2025, attributed by the site operator to failure at low ambient temperature; the second spans November 2025 to March 2026 and followed the failure of the battery, which had been in service since installation and was not replaced during the deployment. Both fall in winter, so the mountain deployment carries almost no cold-season data: after quality control the winter characterization rests in December 2024 alone, with 82 valid daytime records, and should be read as indicative only. Neither interruption is attributable to the harvesting source, which continued to produce normally in both cases.

**Instrumental drift.** The 42% loss of sensor response is the most consequential limitation of the campaign, since it affects the headline site statistics and was not identified during operation. It was detected retrospectively and only because a second radiometric channel happened to be telemetered for an unrelated reason; without it, the decline would have been indistinguishable from a genuine change in the local irradiance regime.

**Data completeness.** Delivery ratios differ substantially between sites (96.8%, 83.5% and 58.2% for the rural, mountain and urban deployments), so the three records differ in density as well as length, and the urban record is both the shortest and the sparsest. Records lost in transmission are unrecoverable, as the nodes do not buffer.

**Temporal site overlap:** The three stations never operated at the same time, with AWS2-R from May to August 2024, AWS1-M from August 2024 to October 2025, and AWS1-U from March to May 2026. Therefore, differences between the sites cannot be attributed solely to location, as inter-annual and seasonal meteorological variability is also included in them. The higher mean at Trnava (351.5 W/m^2^, compared to 267.7 W/m^2^ at Ovčar) is partly a reflection of the fact that Trnava only measured during the high-irradiance summer; whereas, the Ovčar record includes a full annual cycle.

**The disparity in sample size:** The number of valid daytime records differs considerably (16,971 for AWS1-M, 7248 for AWS2-R, 1926 for AWS1-U), purely due to the different deployment durations, since all stations were sampled at 10 min intervals. The 55-day urban record only restricts the statistical weight of any conclusion for AWS1-U and does not permit meaningful quantification of uncertainty for that site.

**Availability of reference data:** The analysis was performed when the NASA POWER archive only extended to 31 March 2026, meaning there is no concurrent satellite reference for AWS1-U for the months of April and May 2026. This is addressed by the model-based reconstruction of Section 5.3 based on archived data from March 2026. Residual uncertainty includes the extrapolation model error (holdout RMSE of 0.506 kWh/m^2^/day for all-sky irradiance) and the inter-annual variability that the model cannot capture. A near match of reconstruction (329.6 W/m^2^) and previous-year proxy (330.3 W/m^2^) suggests that this uncertainty does not materially affect the urban attenuation estimate, which is in any case dominated by the 12:1 obstruction ratio.

**Absence of independent calibration.** No station was cross calibrated against a certified reference pyranometer, so the absolute accuracy of the reported irradiance rests on manufacturer specifications and literature values [29,30,35].

Hence, conclusions from inter-site comparisons are subject to an inherent uncertainty due to the lack of co-located measurements at the same time. To rigorously attribute the observed differences to site characteristics, we would need to have identical nodes at all sites for at least 12 months. The present data set is interpreted as a first, exploratory characterization of the regional irradiance environment. Continued measurements at all sites until 2026–2027, combined with the network extension planned, should provide the concurrent data for a proper attribution analysis.

Subject to these reservations, the dataset is, to the best of the authors’ knowledge, the first multi-site ground-based IoT irradiance record for central Serbia across contrasting terrain types [31] and provides a usable empirical basis for regional solar resource work and standard model development.

## 7. Conclusions

In this paper we present ground-based solar irradiance measurements from a low-cost IoT network at three rather different sites in the Čačak region of Serbia. The main results are as follows. All three sites are within the same NASA POWER grid cell [1] but have very different irradiance regimes, with mean values of 267.7, 351.5 and 19.6 W/m^2^ for the mountain, rural and urban site respectively; this directly demonstrates how much coarse satellite products miss in the local resource assessment. Of the three ground references, the most reliable is the rural Trnava (R^2^ = 0.567 against NASA POWER). The mountain Ovčar has a systematic negative bias (MBE = −138.89 W/m^2^) due to ridge shading and convective cloud. The urban site gets around 6% of the reference, owing to obstruction by buildings and vegetation. This shows the challenge of putting representative sensors in dense urban fabric. Finally, the physics-based firmware conversion [30,35] implemented in the BH1750 nodes produced physically consistent absolute values at all sites, validating the sensor system design.

The regression modeling of the long-term NASA POWER series confirmed, on local data, the established ranking, with XGBoost first (R^2^ = 0.998), TCN-GNN as the leading neural architecture (R^2^ = 0.968) and the added fully connected baseline reaching R^2^ = 0.912, which places a plain ReLU network on a par with three-layer recurrent stacks and indicates that sequential modeling contributes little to this dataset.

The deployment analysis validates the integration of the machine learning framework with the embedded sensing hardware. A depth-limited gradient-boosted corrector executes on the ESP32 in 46.3 µs using 11.1 kB of flash (R^2^ = 0.9985), and an INT8 fully connected network runs in 138.1 µs using 3.0 kB (R^2^ = 0.9596), while the TCN-GNN graph hybrid is relegated to the cloud due to sparse tensor memory requirements. Both edge-feasible models consume four orders of magnitude less energy (~6–18 µJ) than a single Wi-Fi transmission (~200 mJ). This justifies a hierarchical intelligence architecture: the ESP32 edge node performs real-time bias correction, residual-based anomaly detection, and transmission gating locally, while the cloud infrastructure manages computationally intensive spatio-temporal modeling, long-term archival, and periodic retraining to deploy updated model parameters back to the field.

In future work the network will be extended, with LoRaWAN uplinks removing the backhaul constraint that currently limits site selection, the acquisition chain will be hardened, certified RHMZ measurements will be included for cross-validation [34], and terrain-aware correction models will be developed for complex mountain environments.

## Figures and Tables

**Table 1:** Link reliability by site deployment, derived from the ingestion log. The delivery ratio is computed on operational days against the 139.6 records per day expected at the realized 619 s interval, so it measures the communication link rather than station availability, which the operational-day count reports separately.

Deployment	Window	Calendar Days	Operational Days	Records	Median Rec/Day	Delivery (%)	Gaps > 1 h	Max Gap (d)
AWS1-M (mountain)	August 2024–October 2025	447	270	31,479	132	83.5	98	98.0
AWS2-R (rural)	May–August 2024	97	88	11,895	139	96.8	8	6.7
AWS1-U (urban)	March–May 2026	92	46	3742	81	58.2	34	8.4

**Table 2:** Photovoltaic harvesting conditions at AWS1-M over the stable operating period (August 2024–May 2025), from the telemetered panel voltage. The daily integral is a relative proxy; the panel current was not measured. December carries too few complete days for a representative integral.

Month	Productive Hours per Day (Median)	Daily Voltage Integral (V·h, Median)	Days with More Than 60 Records
April	10.5	349.6	26
May	11.3	332.6	31
August	11.4	428.3	19
September	10.3	365.3	29
October	9.5	382.5	27
November	3.6	258.2	8
December	1.4	—	—

**Table 3:** AWS station characteristics.

Station	Location	Coordinates	Elevation (m)	Period	Site Type
AWS1-M	Ovčar (Dučalovići)	43.8887° N, 20.2178° E	~550	August 2024–October 2025	Mountain
AWS1-U	Čačak city center	43.8896° N, 20.3521° E	~230	March–May 2026	Urban
AWS2-R	Trnava	43.8507° N, 20.3746° E	~280	May–August 2024	Rural

**Table 4:** Monthly midday response of the irradiance sensor and of the photovoltaic panel at AWS1-M, as 95th percentiles (p95) of the 10–14 h window on quality-controlled records.

Month	Irradiance p95 (W/m^2^)	Panel Voltage p95 (V)	N
August 2024	820	68.9	545
September 2024	838	73.7	736
October 2024	829	76.2	659
April 2025	772	73.7	682
May 2025	815	73.7	849
June 2025	743	69.4	559
July 2025	684	63.1	598
August 2025	616	59.2	543
September 2025	534	51.7	407

**Table 5:** Data quality control summary.

Station	Raw Records	After Nighttime Filter	After Plausibility Filter	Final Valid Records	Retention %
AWS1-M + AWS1-U (AWS1 total) *	35,221	21,847	16,971 + 1926	18,897	53.7%
AWS2-R (Trnava)	11,895	8103	7248	7248	60.9%

* AWS1 total covers both the mountain (August 2024–October 2025) and the urban (March–May 2026) deployment periods.

**Table 6:** Summary of regression performance by model family on the Čačak monthly NASA POWER series, evaluated on the held-out chronological test split.

Model	R^2^ (Test)	RMSE	Family	Edge Feasibility
XGBoost	0.998	0.093	Gradient boosting	Yes, depth-limited
TCN-GNN	0.968	0.345	Graph hybrid	No, not convertible
LSTM-3	0.917	0.577	Recurrent	Not evaluated
ANN-ReLU	0.912	0.640	Fully connected	Yes, INT8
CNN-3	0.906	0.618	Convolutional	Not evaluated
TCN-1	0.863	0.743	Temporal convolutional	Not evaluated
TCN-recurrent hybrids	−0.020 to −0.004	≈2.05	Divergent	n/a

**Table 7:** Reference values for the AWS1-U window: March 2026 (archived) and April–May 2026 (modeled).

Month	ASSSDI (kWh/m^2^/Day)	CSSSDI (kWh/m^2^/Day)	Daytime-Avg GHI (W/m^2^)	Source
March 2026	3.280	5.021	280.5	NASA POWER archive
April 2026	4.497	6.702	340.1	Seasonal model
May 2026	5.356	7.780	368.2	Seasonal model

**Table 8:** Measured on-node cost of the converted models on the ESP32-D0WD-V3 at 240 MHz, mean over 1000 repetitions with the 0.601 µs timing overhead subtracted. Energy per inference was not measured; the estimates quoted in the text are derived from the datasheet supply current and the measured execution time.

Model	Flash (B)	RAM (B)	Latency (µs)	Range (µs)	R^2^ After Conversion	Structure
XGBoost, depth 3, 40 + 40 trees	11,140	24	46.3	46–73	0.9985	1098 nodes
Fully connected 72-32-16-2, INT8	3048	480	138.1	138–143	0.9596	2898 parameters
TCN-GNN	—	—	—	—	—	not convertible

**Table 9:** Descriptive statistics of solar irradiance by site.

Station	Mean (W/m^2^)	Median (W/m^2^)	Max (W/m^2^)	Std (W/m^2^)	% of NASA POWER
AWS1-M Ovčar, full period	267.7	147.0	990.0	260.8	~81%
AWS1-M Ovčar, stable period (August 2024–May 2025)	302.0	207.0	990.0	266.4	~92%
AWS2-R Trnava	351.5	282.0	1069.0	285.0	~107%
AWS1-U Čačak	19.6	8.0	78.0	21.3	~6%
NASA POWER	329.5	—	989.1	260.7	100%

**Table 10:** Statistical performance of AWS stations vs. NASA POWER.

Station	N	RMSE (W/m^2^)	MAE (W/m^2^)	MBE (W/m^2^)	R^2^	Note
AWS1-M Ovčar	3072	240.74	193.21	−138.89	0.489	Full period
AWS1-M Spring	774	192.16	151.95	−105.21	0.592	March–May
AWS1-M Summer	1358	286.63	239.84	−218.10	0.566	June–August
AWS1-M Autumn	912	202.87	162.19	−52.84	0.451	September–November
AWS2-R Trnava	1202	212.69	178.04	−94.52	0.567	Full period
AWS1-U Čačak	N/A	N/A	N/A	~−500	N/A	Descriptive only

## Data Availability

The quality-controlled measurement records for all three sites, the retrieval script for the NASA POWER reference, the training and evaluation code for all 25 model configurations, the conversion scripts and generated headers for the on-node models, and the benchmark sketch used for the measurements are available at https://github.com/dejanvujicic/cacak-irradiance-repo. The complete dataset and implementation codebase supporting this study are publicly available on GitHub at https://github.com/shrihari808/cacak-irradiance. The repository includes: (1) raw and quality-controlled ground measurement records for all three sensor deployments (mountain, rural, and urban); (2) the 25-year monthly and hourly NASA POWER satellite data alongside MissForest imputation scripts; (3) source code and trained PyTorch and Scikit-Learn/XGBoost model checkpoints for all 24 initial regression configurations and the added ANN-ReLU baseline; (4) model conversion scripts and generated C header files for ESP32 on-device deployment; and (5) the complete ESP32 benchmarking sketch and reproducibility instructions for independent verification.

## Abbreviations

The following abbreviations are used in this manuscript:

ANN	Artificial Neural Network
ASICI	All-Sky Insolation Clearness Index
ASSA	All-Sky Surface Albedo
ASSSDI/CSSSDI	All-Sky/Clear-Sky Surface Shortwave Downward Irradiance
AWS	Automatic Weather Station
BIPV	Building-Integrated Photovoltaics
CNN	Convolutional Neural Network
GHI	Global Horizontal Irradiance
GNN	Graph Neural Network
GRU	Gated Recurrent Unit
I2C	Inter-Integrated Circuit bus
INT8	8-bit integer quantization
LoRaWAN	Long Range Wide Area Network
LSTM	Long Short-Term Memory
MBE	Mean Bias Error
MSE/MSLE	Mean Squared (Logarithmic) Error
NVS	Non-Volatile Storage
OTA	Over-The-Air update
POWER	Prediction Of Worldwide Energy Resources
RHMZ	Republic Hydrometeorological Service of Serbia
RMSE/RMSLE	Root Mean Squared (Logarithmic) Error
RNN	Recurrent Neural Network
SVF	Sky View Factor
TCN	Temporal Convolutional Network
TLS	Transport Layer Security
WSN	Wireless Sensor Network
XGBoost	Extreme Gradient Boosting
MAE/MAPE	Mean Absolute (Percentage) Error

## Appendix A

Here we briefly describe the machine learning and deep learning architectures used to model the regression of the long-term satellite series, and the metrics used to evaluate them.

### Appendix A.1. Extreme Gradient Boosting (XGBoost)

Gradient boosting is an ensemble method for combining weak learners to form a strong learner. We train each new model to minimize the loss of the previous one, using the gradient descent method. The basic gradient boosting regressor starts with a constant prediction for all samples, usually the mean of the target, then sequentially builds regression trees, where each tree corrects the errors of the previous trees.

A further development of this idea is XGBoost [9]. It adds second-order gradient optimization, regularization and tree pruning compared to the traditional gradient boosting regressor, making it fast, accurate and less likely to overfit complex data such as time series. Boosting starts with an initial prediction, often the mean of target values, and then sequentially adds new learners to reduce the loss. The model is constructed with the following formulation:

(A1) $$f\left(x\right)=\sum_{i=1}^{M}f_{i}(x),\;f_{i}\in F,$$

Here, *F* is the set of all possible regression trees and *M* is the number of boosting rounds. Each function is chosen so that the regularized loss is minimized:

(A2) $$L\left(f\right)=\sum_{k=1}^{N}L\left(y_{k},\hat{y_{k}}\right)+\sum_{i=1}^{M}\Omega \left(f_{i}\right),$$

(A3) Ωf=γT+12λ∑jwj2,

where *L* is the loss function, *Ω*(*f_i_*) is the regularization term which penalizes complexity, *T* is the number of leaves in the tree, *w_j_* is the weight of each leaf, and γ and λ are the regularization hyperparameters. XGBoost uses a second-order Taylor expansion of the loss, so for each training instance it computes the gradient *g_k_* and the hessian *h_k_* of the loss at the current prediction, which makes the training faster and more accurate.

### Appendix A.2. Long Short-Term Memory

The long short-term memory (LSTM) network [10] is a recurrent neural network which solves the vanishing and exploding gradient problems in sequence learning. It is one of the most used models for time series forecasting, with applications in speech recognition, demand prediction and renewable energy forecasting. The LSTM captures both short-term fluctuations and long-term dependencies in sequential data; its central element is a memory cell whose content is controlled by gating mechanisms across the time steps.

The memory cell is regulated by the input, forget and output gates. The input gate adds useful information to the memory, the forget gate removes the parts of the past information which are not needed any more, and the output gate decides what is passed to the next step. In this way the LSTM keeps the relevant information and filters out the noise. The gates are realized with sigmoid and tanh activation functions which update the hidden and cell states.

In contrast to ARIMA or VARIMA, which are limited to linear assumptions, LSTM can learn nonlinear and multivariate dependencies. An LSTM-based multivariate framework showed, for example, good results in tourism demand prediction, where weather conditions, holidays and events were used as additional inputs [45]. The price for this flexibility is sequential processing: each prediction depends on the previous step, so the training on very long sequences is slow. Extensions such as bidirectional LSTMs and attention mechanisms further improve accuracy and stability.

### Appendix A.3. Gated Recurrent Unit

The gated recurrent unit (GRU) [11] is a recurrent network for sequence modeling, applied in speech recognition, language modeling and time series forecasting. It contains a reset gate and an update gate: the reset gate controls how much of the past information is discarded when the current activation is computed, and the update gate balances between keeping the old information and adding the new input. With these two gates the GRU preserves long-term dependencies and at the same time adapts quickly to short-term changes. The gates use sigmoid functions, and the candidate activation uses tanh.

Compared with the LSTM, the GRU has fewer trainable parameters, needs less memory and trains faster. Several studies showed that GRUs reach the same or better accuracy than LSTMs in many time series tasks, particularly on smaller datasets or with limited resources [46]. Unlike temporal convolutional networks, the GRU processes the input sequentially, which prevents full parallelization on very long sequences, but the smaller number of parameters compensates for this. Modern variants combine GRUs with convolutional or attention layers to capture both local features and long-range dependencies, and the GRU naturally integrates several input features, such as temperature, humidity, wind speed and past irradiation values.

### Appendix A.4. One-Dimensional Convolutional Networks

One-dimensional convolutional networks (1D CNN) process sequential and time-series data efficiently. A convolution is a mathematical operation which combines two functions into a third one; in a 1D CNN, a kernel (filter) with learned weights slides over a one-dimensional array. At every position a dot product is computed, and the result is a feature map which contains information about the patterns present in the data, so the features are extracted automatically, without manual feature engineering. The main advantage of 1D CNNs is the small number of parameters compared with fully connected layers. Convolutional layers are usually stacked and combined with pooling layers into deeper architectures; the convolution kernels learn from the training data and process the test data through convolution and pooling into a new feature space [47].

Pooling reduces the dimensionality of the feature maps and keeps the important information. The tuning of a 1D CNN includes the kernel size, which defines the receptive field of each filter (several kernel sizes can be used in parallel to capture patterns on different scales), and the number of filters, which defines the capacity of the model. 1D CNNs are used to find patterns such as peaks or troughs in time series, and they have been applied many times to financial series, electricity load forecasting and solar energy prediction [48].

### Appendix A.5. Temporal Convolutional Networks

Temporal convolutional networks (TCN) are convolutional networks specialized for sequence modeling, an alternative to recurrent networks such as the GRU and LSTM. The main design principle which separates the TCN from a traditional CNN is that the output sequence has the same length as the input sequence; in the simplest description, a TCN is a 1D fully convolutional network with causal convolutions. The causal convolutions guarantee that the output at time *t* depends only on the inputs from time *t* and earlier, which keeps the temporal order needed for prediction. The equal input and output lengths are obtained with zero padding of length (kernel size − 1).

A simple causal convolution can look back only over a history whose size grows linearly with the network depth. TCNs therefore use dilated convolutions [17], which insert gaps between the filter elements and enlarge the receptive field without additional parameters. The dilation factor *d* defines the spacing between the filter elements, and when the dilation grows across the layers, the receptive field grows exponentially. In this way the network sees a much larger part of the input without the cost of larger kernels [49].

Since the same filter is used in every layer, the TCN processes the whole sequence in parallel, both in training and in inference, while in an RNN each prediction must wait for the previous one. TCNs also need less memory than gated RNNs, because the filters are shared across the layers, and they are more stable with respect to gradients: the backpropagation path does not follow the temporal direction of the sequence, so the exploding and vanishing gradients typical for RNNs do not appear, and no special gating is needed. On the other hand, during inference the TCN must keep the raw sequence up to the effective history length, while an RNN keeps only a fixed-size hidden state. Modern implementations often use several parallel dilated convolutions with different dilation rates, attention layers, or combinations with other architectures.

### Appendix A.6. Hybrid Models

The LSTM-GRU model connects the memory capabilities of the LSTM with the computational efficiency of the GRU in a sequential arrangement: the LSTM first captures the long-term dependencies and the more complex temporal patterns, and the GRU units then provide additional temporal modeling at a lower cost. Two variants were used in this study, one with a single LSTM layer followed by a single GRU layer, and a deeper one with one LSTM layer followed by two GRU layers; both end with fully connected layers which produce the regression output.

The CNN-LSTM model connects a convolutional network and an LSTM in series. The CNN part extracts the spatial features and patterns from the input through convolutional and max-pooling layers, and the LSTM then models the temporal sequence of these features and handles the long-term dependencies. The two variants used here share the initial Conv1D layer and a max-pooling layer; the first has a single LSTM layer which returns a summary vector, and the second has two stacked LSTM layers for a more hierarchical processing.

The CNN-GRU architecture follows the same pattern, but with GRU layers for the temporal part. The CNN extracts the spatial features through convolutions, activations and max pooling, and the GRU—as a simpler alternative to the LSTM (the forget and input gates are merged into one update gate, and the cell state is merged with the hidden state)—models the temporal dependence with fewer parameters. Fully connected and dropout layers regularize the final predictions [50].

The TCN-GRU model joins the temporal convolutional network with gated recurrent units, so the efficiency of dilated convolutions is combined with the simple recurrent processing of the GRU. The TCN part captures the spatiotemporal features through dilated causal convolutions, without manual feature engineering, and the GRU layers learn the temporal dependencies at a lower cost than the LSTM; the dilations and residual connections keep the information from the more distant past. In both variants used here, a TCN layer first processes the whole input sequence as a feature extractor; the first variant sends these features to one GRU layer, the second to two stacked GRU layers, and both end with a dense feed-forward network which produces the regression output [51].

### Appendix A.7. Evaluation Metrics

Since the modeling problem is a regression task, the following metrics were chosen. In the formulas, *a_i_* is the actual value of the *i*-th observation, *b_i_* the predicted value, *m* the number of observations, and *j* the number of independent variables.

Mean Absolute Error (MAE) is the average absolute difference between the actual and predicted values:

(A4) MAE=1m∑i=1mai−bi.

Mean Absolute Percentage Error (MAPE) is the average of the absolute percentage difference between the actual and the predicted values. The advantage of MAPE is that it does not have any units:

(A5) MAPE=1m∑i=1mai−biai×100.

Mean squared error (MSE) is the mean of the squared differences between the actual and the predicted values. Since the difference is squared, it is very sensitive to outliers and imbalances in the data:

(A6) MSE=1m∑i=1mai−bi2.

Root mean squared error (RMSE) is the square root of the MSE. It is sensitive to outliers, but to a lesser extent than the MSE:

(A7) RMSE=1m∑i=1mai−bi2.

Mean Squared Logarithmic Error (MSLE) is the average of the squared difference between the logarithm of the actual values and the logarithm of the predicted values. It is useful when more importance should be given to errors where predictions are too low compared to the actual values, since the logarithm reduces the impact of large differences for higher values:

(A8) MSLE=1m∑i=1mlnai+1−lnbi+12.

Root Mean Squared Logarithmic Error (RMSLE) is the square root of the MSLE. Since the logarithmic difference is squared, it is less sensitive to large differences for higher values than the RMSE:

(A9) RMSLE=1m∑i=1mlnai+1−lnbi+12.

The coefficient of determination (R^2^) measures the ratio of the variance explained by the model to the total variance of the target variable:

(A10) $$R^{2}=1-\frac{\sum_{i=1}^{m}{\left(a_{i}-b_{i}\right)}^{2}}{\sum_{i=1}^{m}{\left(a_{i}-\bar{a}\right)}^{2}}.$$

Adjusted R^2^ is a modified version of R^2^ that considers independent variables that do not significantly improve the regression model’s performance. Its value decreases if unnecessary predictors are included that do not improve the model’s fit:

(A11) $$Adjusted\;R^{2}=1-\frac{\left(1-R^{2}\right)\left(m-1\right)}{m-j-1}.$$

## Appendix B

**Table A1:** Performance of all 25 regression model configurations on the Čačak monthly NASA POWER series (2001–2026), evaluated on the held-out chronological test split and sorted by coefficient of determination. The ANN-ReLU row is added and is trained under the protocol described in Section 4. Targets are the all-sky and clear-sky surface shortwave downward irradiance, reported jointly.

Model	MAE	MAPE (%)	MSE	RMSE	MSLE	RMSLE	R^2^	Adj R^2^
XGBoost	0.0694	1.80	0.0086	0.0926	0.0003	0.0184	0.9978	0.9976
TCN-GNN	0.2214	6.06	0.1193	0.3454	0.0042	0.0651	0.9683	0.9671
LSTM-3	0.4509	11.88	0.3329	0.5769	0.0142	0.1190	0.9172	0.9142
LSTM-GRU-1	0.4413	11.53	0.3388	0.5821	0.0142	0.1193	0.9162	0.9132
ANN-ReLU	0.5242	14.38	0.4094	0.6399	0.0157	0.1252	0.912	0.9073
LSTM-1	0.4797	12.18	0.3654	0.6045	0.0144	0.1199	0.9096	0.9064
LSTM-2	0.4805	12.58	0.3674	0.6061	0.0157	0.1252	0.9089	0.9057
GRU-1	0.4833	12.50	0.3755	0.6128	0.0142	0.1192	0.9067	0.9034
GRU-2	0.4981	13.38	0.3801	0.6165	0.0148	0.1218	0.9061	0.9028
CNN-3	0.4969	14.81	0.3823	0.6183	0.0179	0.1337	0.9057	0.9023
GRU-3	0.4783	11.98	0.3855	0.6209	0.0143	0.1198	0.9040	0.9005
LSTM-GRU-2	0.5395	13.59	0.4417	0.6646	0.0169	0.1301	0.8901	0.8862
CNN-GRU-1	0.5308	14.30	0.4481	0.6694	0.0182	0.1349	0.8893	0.8853
CNN-GRU-2	0.5496	13.99	0.5081	0.7128	0.0199	0.1409	0.8744	0.8699
CNN-LSTM-1	0.5817	16.34	0.5402	0.7350	0.0229	0.1512	0.8670	0.8623
CNN-LSTM-2	0.5215	13.11	0.5368	0.7327	0.0205	0.1432	0.8670	0.8622
TCN-1	0.5590	13.76	0.5526	0.7434	0.0184	0.1356	0.8629	0.8580
CNN-2	0.6084	18.73	0.5916	0.7691	0.0280	0.1675	0.8566	0.8515
TCN-2	0.6031	16.65	0.6174	0.7857	0.0283	0.1683	0.8476	0.8422
TCN-3	0.8688	26.85	1.1357	1.0657	0.0494	0.2224	0.7261	0.7163
CNN-1	1.4180	33.85	3.0034	1.7330	0.0944	0.3072	0.2789	0.2531
TCN-LSTM-2	1.8012	55.90	4.2046	2.0505	0.1655	0.4068	−0.0036	−0.0395
TCN-GRU-1	1.8010	54.77	4.2268	2.0559	0.1639	0.4049	−0.0094	−0.0455
TCN-LSTM-1	1.8014	54.17	4.2444	2.0602	0.1633	0.4041	−0.0140	−0.0502
TCN-GRU-2	1.8050	53.52	4.2704	2.0665	0.1626	0.4032	−0.0202	−0.0566
